# Selective Anticervical Cancer Injectable and Self-Healable Hydrogel Platforms Constructed of Drug-Loaded Cross-Linkable Unimolecular Micelles in a Single and Combination Therapy

**DOI:** 10.1021/acsami.4c01524

**Published:** 2024-03-15

**Authors:** Monika Gosecka, Mateusz Gosecki, Piotr Ziemczonek, Malgorzata Urbaniak, Ewelina Wielgus, Monika Marcinkowska, Anna Janaszewska, Barbara Klajnert-Maculewicz

**Affiliations:** † Centre of Molecular and Macromolecular Studies, Polish Academy of Sciences, Sienkiewicza 112, 90-363 Lodz, Poland; ‡ Department of General Biophysics, Faculty of Biology and Environmental Protection, University of Lodz, 141/143 Pomorska Street, 90-236 Lodz, Poland

**Keywords:** selective anticervical cancer therapy, self-healable, injectable hydrogel, hydrophobic drug, drug carrier

## Abstract

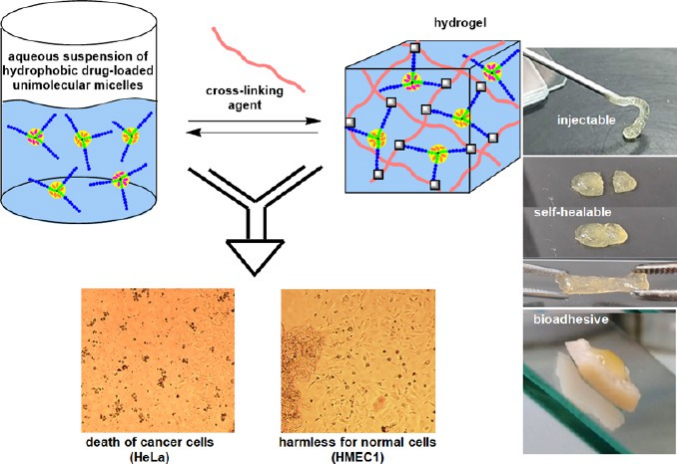

In the face of severe side effects of systemic chemotherapy used in cervical cancer, topical selective drug carriers with long-lasting effects are being sought. Hydrogels are suitable platforms, but their use is problematic in the case of delivery of hydrophobic drugs with anticancer activity. Herein, hydrogels constructed of unimolecular micelles displaying enhanced solubilization of aromatic lipophilic bioactive compounds are presented. Star-shaped poly(benzyl glycidyl ether)-*block*-poly(glycidyl glycerol ether) with an aryl-enriched core show high encapsulation capacity of poor water-soluble nifuratel and clotrimazole. Nifuratel attained selectivity against cervical cancer cells, whereas clotrimazole preserved its original selectivity. The combination of unimolecular micelles loaded with both drugs provided synergism; however, they were still selective against cervical cancer cells. The cross-linking of drug-loaded unimolecular micelles via dynamic boronic esters provided injectable and self-healable hydrogel drug carriers also displaying synergistic anticancer activity, suitable for intravaginal administration and assuring the effective coverage of the afflicted tissue area and efficient tissue permeability with hydrophobic bioactive compounds. Here, we show that the combination of star-shaped polyether amphiphiles and boronic ester cross-linking chemistry provides a new strategy for obtaining hydrogel platforms suitable for efficient hydrophobic drug delivery.

## 1 Introduction

Cervical cancer, a malignant epithelial tumor that forms in the uterine cervix,^1^ is one of the most common causes of death among women worldwide.^2^ Chemotherapy is one of the main ways of cervical cancer treatment. The use of a single therapeutic agent is not effective in the treatment of cancer, and therefore combinatorial therapy is often necessary.^3^ Various combinations of drugs such as cisplatin, paclitaxel, bevacizumab, carboplatin, topotecan, and gemcitabine given intravenously are being recommended as first-line therapies.^4,5^ The problem with conventional chemotherapy is, however, the lack of selectivity toward cancer cells, which means that both cancer cells and normal cells are killed in parallel. Although these drugs have been approved by the FDA for the treatment of cervical cancer,^6^ they cause serious side effects.^7−10^ For example, after applying the high-dose chemotherapy for treatment, often a subsequent bone marrow transplant is required.^11^ Due to the high toxicity of drugs used in chemotherapy, it is essential to include supportive treatment, i.e., protection against the side effects of chemotherapy.^12^ Furthermore, in addition to the lack of chemotherapeutics selectivity against cancer cells, cancer cells acquire the ability to evade the effect of applied drugs, which reduces the effectiveness of cancer treatment.^13−17^ It is now desirable to search for new substances with anticancer effects or to elaborate drug carriers assuring the reduction of the negative effects of the drugs used for cancer treatment.^18^ Local therapy by the drug administration directly on the afflicted site can avoid systemic side effects and thus is a valuable approach. The usage of aqueous drug-loaded colloids, however, is not efficient due to the quick and uncontrolled leakage that reduces the efficiency of therapy.^19^ To prolong the contact of the drug with the diseased tissue to increase the efficiency of the therapy and to avoid drug resistance, hydrogel carriers of active substances seem to be the most promising candidates for intravaginal applications. However, several parameters are required, such as drug solubility, injectability, bioadhesion under wet conditions, controlled drug release rate, and rheological properties to ensure that the hydrogel platform is retained at the target site and the carrier is completely removed once it has served its purpose. To address the problem of high toxicity of currently applied drugs, for the study, we applied drugs that are routinely applied in vulvovaginitis therapies, such as nifuratel^20,21^ and clotrimazole,^22,23^ and for which anticancer activity has been recently reported. Marinho-Carvalho et al. showed that clotrimazole inhibits cellular glycolysis by direct inhibition of phosphofructokinase EC 2.7.1.11—the key regulatory enzyme of the glycolytic pathway and a CaM-binding protein.^23^ Compared to normal cells, cancer cells have an increased rate of glycolysis,^24^ and their mitochondria metabolize (in addition to pyruvate), which allows for faster biosynthesis of lipids and amino acids, necessary for building membranes and proteins.^25^ This glycolytic preference of cancer cells is called the Warburg effect.^26^ Clotrimazole targets the Warburg effect, which means that it targets cancer cells without affecting normal cells.^27^

The antitumor activity of nifuratel most likely involves inhibition of a signal transducer and an activator of transcription 3 (STAT3).^20^ In addition, it may block interleukin-6-induced activation of the STAT3 signaling pathway, hence inhibiting tumor cell proliferation, inducing apoptosis, and reducing induced apoptosis, and reduce the growth of gastric cancer cells.^20^ On the contrary to clotrimazole, the anticancer activity of nifuratel, until now detected only against human gastric cancer cells, is not selective, as it affects both normal and cancer cells.^20,21^

The low solubility of both clotrimazole and nifuratel in water is, however, a main obstacle in their usage in chemotherapy.^28^ The use of hydrogels as carriers for water-insoluble drugs is also problematic, as an effect of the significant difference between the hydrophilic nature of the hydrogel structure and the hydrophobic character of the selected bioactive compounds.

This problem can be, however, overcome by proper design of the hydrogel structure, i.e., by the incorporation of drug-loaded particles^29^ or the network construction based on copolymers displaying critical micelle concentration.^30^ In the latter case, the network stability is, however, strictly dependent on the copolymer concentration or applied shear force.^31^ The usage of single-molecular amphiphilic core–shell structures, i.e., unimolecular micelles, which are intrinsically stable, retaining their structure regardless of the concentration,^32^ can, however, overcome the instability problem of standard micellar-based self-assemblies. The construction of unimolecular micelles with a defined hydrophobic core suitable for encapsulation of hydrophobic active substances and a hydrophilic corona equipped with functional groups can provide not only increased solubility of drugs in aqueous media but also the possibility of the hydrogel formation. In this work, we report the hydrophobized hydrogels constructed of nonlinear polyether-based three-arm unimolecular micelles composed of a poly(benzyl glycidyl ether) core and different lengths of a poly(glyceryl glycerol ether) shell, PBGE-PGGE cross-linked reversibly by boronic ester cross-links. Unexpectedly, PBGE-PGGE-based unimolecular micelles endow the selectivity of nifuratel toward the HeLa cell line, which is highly beneficial in view of the treatment of cervical cancer. In addition, the encapsulation of clotrimazole within PBGE-PGGE amphiphiles did not reduce its anticervical cancer selectivity. A combinatory therapy based on the usage of low doses of both nifuratel and clotrimazole encapsulated within unimolecular micelles both in the aqueous solution and upon their incorporation into the hydrogel network has shown a significant synergism in the anticancer activity simultaneously along with maintaining the selectivity toward cancer cells. This study shows the dependence amongst the structural features of amphiphilic star-shaped copolyethers, the encapsulation efficiency of hydrophobic drugs, and the selectivity action of drugs in both a single and combination therapy in the aqueous medium and the rheological properties of hydrogels obtained. This work establishes a versatile hydrogel platform as a suitable carrier of poorly water-soluble bioactive substances equipped with aromatic groups.

## 2 Experimental Section

### 2.1 Materials

1,1,1-Tris(hydroxymethyl)propane (Sigma-Aldrich) was dissolved in acetone and precipitated by addition of diethyl ether. Then, 60 wt % NaH in mineral oil (Merck) was washed with dry tetrahydrofuran (THF) to remove oil and dried. The oil was removed by washing with dry tetrahydrofuran, and then drying under reduced pressure. Benzyl glycidyl ether (BGE, Sigma-Aldrich) was dried over CaH_2_ and then distilled under reduced pressure before use.

d,l-1,2-Isopropylidene glyceryl glycidyl ether (IGG) was synthesized according to the procedure described by Wurm et al.^33^ THF (Sigma-Aldrich) was dried over a Na/K alloy. Dimethyl sulfoxide (DMSO, Sigma-Aldrich) was purified and dried according to the procedure described in ref (34) and stored over 4 Å molecular sieves.

Nifuratel (TCI) and clotrimazole (Thermoscientific) were used as received without any further purification. A Strat-M membrane was used as a nonanimal model for transdermal diffusion testing to predict the diffusion of drugs through human skin.

The simulated vaginal fluid (SVF) of pH = 4.2 was prepared according to the composition described in ref (35).

### 2.2 Synthesis of 3-*star*-[Poly(benzyl glycidyl ether)-*block*-poly(glyceryl glycerol ether)] (PBGE-PGGE)

NaH (32 mg, 0.8 mmol) was used to convert 1,1,1-tris(hydroxymethyl)propane (106.1 mg, 0.8 mmol) in dry tetrahydrofuran into the alcoholate form. The reaction mixture was stirred for 24 h at 50 °C under a vacuum. Then, tetrahydrofuran was distilled, and the alcoholate was dissolved in 2 mL of DMSO. In the next step, benzyl glycidyl ether (BGE) (6.568 g; 40 mmol) was introduced into the reactor (a Schlenk flask) with the in situ-generated anionic polymerization initiator. The Schlenk flask was placed in an oil bath at 50 °C for 8 h under a vacuum. After this, the flask content was dialyzed against DMSO for 48 h using a dialysis bag (Prewetted RC Tubing MWCO = 1 kDa, Spectra/Por6), changing the solvent twice per day. The polymerization yield was equal to 90%. The PBGE homopolymer was characterized based on the ^1^H NMR spectrum and gel permeation chromatography (GPC).

^1^H NMR (400 MHz, DMSO-*d*_6_, δ, ppm): 7.21 (m), 4.70 (OH end groups), 4.35 (m), 3.50 (m), 1.21 (t), 0.67 (d).

Into a Schlenk flask containing a purified and dried portion of poly(benzyl glycidyl ether) (1.35 g; 0.18 mmol), NaH (21.2 mg; 0.53 mmol) was introduced to generate alcoholate in dry tetrahydrofuran for 24 h at 50 °C. After this, tetrahydrofuran was removed by distillation, and an IGG comonomer was introduced. A new portion of dry tetrahydrofuran was then distilled into the Schenk flask. The polymerization was carried out under a vacuum at 80 °C for 5 days. The reaction mixture was diluted with DMSO and transferred to a dialysis bag (Prewetted RC Tubing MWCO = 1 kDa, Spectra/Por6), dialyzing for 48 h by changing the solvent twice a day. After solvent removal, the structure of the 3-*star*-[poly(benzyl glycidyl ether)-*block*-poly(d,l-1,2-isopropylidene glyceryl glycidyl ether)] (PBGE-PIGG) copolymer was confirmed by ^1^H NMR, ^1^H DOSY NMR, and GPC. The polymerization yield was approximately 90%.

^1^H NMR (400 MHz, DMSO-*d*_6_, δ, ppm): 7.21 (m), 4.70 (OH end groups), 4.35 (m), 4.15 (m), 3.97 (m), 3.61 (m), 3.50 (m), 1.31 (s), 1.26 (s), 0.67 (d).

The resulting copolymer was dissolved in methanol and treated with 1 M hydrochloric acid, reaching a pH of approximately 3. The solution was stirred for 3 h at 20 °C to deprotect the 1,2-diol groups, which was then verified by ^1^H NMR spectroscopy. A saturated Na_2_CO_3_ solution was then added, reaching pH = 11, and dialyzed in a dialysis bag (Prewetted RC Tubing MWCO = 1 kDa, Spectra/Por6) against deionized water. After solvent removal, the structure of the synthesized PBGE-PGGE copolymer was analyzed by ^1^H NMR spectroscopy.

^1^H NMR (400 MHz, DMSO-*d*_6_, δ, ppm): 7.21 (m), 4.62–4.49 (OH groups), 4.39 (m), 3.50 (m), 1.26 (s), 0.67 (d).

A series of PBGE-PGGE copolymers with a constant number of core repeating units (DP*_n_* = 15), differing in the length of the hydrophilic segment, were obtained (Table 2).

### 2.3 Synthesis of Poly(acrylamide-*ran*-2-acrylamidephenylboronic acid) (P(AM-*ran*-2-AAPBA))

A P(AM-*ran*-2-AAPBA) copolymer was obtained according to the procedure described previously^36^ using AIBN (0.082 g; 0.5 mmol), acrylamide (4.68 g; 65.84 mmoL), and 2-acrylamidephenylboronic acid pinacol ester (2 g; 7.31 mmoL). The polymerization was carried out in 15 mL of a dimethylformamide (DMF)/dioxane mixture (5:1 v/v) at 70 °C. The copolymer was characterized using ^1^H NMR spectroscopy and GPC. The molar fraction of 2-AAPBA units in the copolymer was equal to 10 mol %, whereas *M*_n_ was 54,000 (*M*_w_/*M*_n_ = 1.21).

### 2.4 Solubilization of Nifuratel within Unimolecular Micelles

A stock solution of nifuratel in methanol at a concentration equal to 0.40 mg/mL was prepared. Samples of the PBGE-PGGE copolymer, 50 mg each, were dissolved in 5 mL of methanol. Different volumes of the nifuratel solution ranging from 2.7 to 31 mL were input to the copolymer solution. The mixtures were stirred for 30 min. Then, methanol was evaporated at 37 °C overnight. The dry polymer-drug content was suspended in 15 mL of deionized water. The suspension was filtered through a 0.45 μm poly(tetrafluoroethylene) (PTFE) syringe filter. The aqueous solution was lyophilized overnight, and the drug loading capacity in PBGE-PGGE micelles was determined by ^1^H NMR spectra using DMF as an internal reference.

### 2.5 Solubilization of Clotrimazole within Unimolecular Micelles

A stock solution of clotrimazole in methanol at a concentration equal to 5 mg/mL was prepared. Then, 100 mg of each copolyether was dissolved in 5 mL of methanol. Different volumes of the clotrimazole solution ranging from 2.7 to 31 mL were added to the methanolic solutions of the copolymer. The mixtures were incubated for 6 h. Then, methanol was evaporated at 37 °C overnight. The dry polymer-drug content was suspended in 15 mL of deionized water. The suspension was filtered using a a 0.45 μm PTFE syringe filter and lyophilized. The drug loading capacity in PBGE-PGGE micelles was determined by ^1^H NMR spectra using DMF as an internal reference.

### 2.6 Nifuratel Release

The study of nifuratel release from drug-loaded PBGE-PGGE copolymers was performed according to the procedure described previously^37^ at pH = 5.5 and 37 °C.

### 2.7 Clotrimazole Release Profile

The study of clotrimazole release from drug-loaded PBGE-PGGE copolymers was performed according to the procedure described previously^36^ at pH = 5.5 and 37 °C.

### 2.8 Hydrogel Formation

Twenty five milligrams of each PBGE-PGGE copolymer was dissolved in 181 μL of deionized water, whereas 42 mg of P(AM-2-AAPBA) was dissolved in 140 μL of deionized water. Then, both solutions were combined, leading to the immediate gelation. The total weight of polymer content was equal to 17 wt %.

### 2.9 Formation of Drug-Loaded Hydrogels

The drug-loaded hydrogels were constructed of PBGE-PGGE macromolecules either loaded with one drug or a mixture of PBGE-PGGE macromolecules loaded with both drugs individually and cross-linked with P(AM-2-AAPBA) in deionized water. However, for the construction of hydrogels equipped with one drug, neat PBGE-PGGE was used to keep the weight fraction of the polymer constant in all investigated hydrogel systems. The concentrations of drugs in the investigated hydrogel systems are given in Table 7.

### 2.10 Scanning Electron Microscopy (SEM) Imaging

Hydrogel samples constructed of PBGE-PGGE, differing in the length ratio of the shell to the core, were frozen and lyophilized. SEM images were recorded on a JEOL JSM 6010LA.

### 2.11 Rheology

The oscillation frequency sweep tests were performed on a Thermoscientific HAAKE MARS 40 rheometer in the linear viscoelastic regime using a parallel plate–plate geometry of an 8 mm diameter with a 0.3 mm gap at 25 and 37 °C. Strain sweep tests of synthesized hydrogels were performed at a frequency of 1 Hz in the range of strain from 0.02 to 2000%. Stress sweep tests for all investigated hydrogel systems were performed at 37 °C at a frequency of 1 Hz. Temperature sweep tests in the range from 10 to 40 °C were carried out at a frequency of 1 Hz using the continuous heating program with a heating rate of 5 °C/min. The self-healing tests for all investigated hydrogels were performed in the oscillation time mode (1 Hz) at 37 °C by monitoring both storage and loss moduli. The hydrogel sample was first placed under a 1% strain for 180 s, and then it was destroyed with a 5 s 300% strain pulse, after which 1% was again applied for sample restoration.

### 2.12 Franz Cell

A hundred milligrams of each hydrogel constructed of drug-loaded PBGE-PGGE with P(AM-2-AAPBA) was placed on a Strat-M membrane into a donor compartment of the Franz cell.

For the study, the hydrogel constructed of PBGE-PGGE_1_CLOT_d and PBGE-PGGE_1_NIF_e contained 8.925 mg of the CLOT/g hydrogel and 0.95 mg of NIF/g, respectively, and the hydrogel based on PBGE-PGGE_2_CLOT_c and PBGE-PGGE_2_NIF_e contained 1.17 mg of the CLOT/g hydrogel and 1.894 mg of NIF/g, respectively. In addition, hydrogels based on PBGE-PGGE_2 were equipped with single drugs at the following concentrations of the hydrogels: 1.17 mg of the CLOT/g hydrogel and 1.894 mg of NIF/g. For comparison, an aqueous suspension of both drugs was prepared in the simulated vaginal fluid at the same drug concentrations, however, adding a neat polymer to keep the weight fraction of the polymer component as well as the cross-linking density of the hydrogels constant.

The receptor compartment was filled with 12.5 mL of the simulated vaginal fluid at 37 ± 1 °C. Aliquots of 0.5 mL of the receptor solution were taken at different time intervals for 168 h. Each withdrawn aliquot was immediately replaced by the same volume of the corresponding fresh portion of the simulated vaginal fluid.

The concentrations of both clotrimazole and nifuratel were determined by an ACQUITY UPLC I-Class chromatography system coupled with a SYNAPT G2-Si mass spectrometer equipped with an electrospray source and a quadrupole-time-of-flight mass analyzer (Waters Corp., Milford, MA). An Acquity BEH_TM_ C18 column (100 × 2.1 mm^2^, 1.7 μm) maintained at 45 °C temperature was used for the chromatographic separation of an analyte. The mobile phase was prepared by mixing 0.1% formic acid (A) and 0.1% formic acid in acetonitrile (B). The elution gradient was: 32% B (0–1.0 min), 32–95% B (1.0–3.0 min), 95–95% B (3.0–3.5 min), 95–32% B (3.5%–3.52 min), and 32–32% B (3.52–7.0 min). The flow rate was 0.45 mL/min, and the injection volume was 0.5 μL for clotrimazole and 2.5 μL for nifuratel.

For mass spectrometric detection, the electrospray source was operated in a positive resolution mode using a capillary voltage of 3.0 kV, a cone voltage of 20 V, a desolvation gas flow of 400 L/h, a temperature of 350 °C, a nebulizer gas pressure of 6.5 bar, and a temperature of the source equal to 100 °C. The mass spectra were recorded in the *m*/*z* range from 100 to 1200. The system was controlled by MassLynx software (Version 4.1), and data processing was performed by the TargetLynxTM program.

The initial stock calibration solutions of clotrimazole and nifuratel were created in the simulated vaginal fluid. The stock solutions were serially diluted with 0.5 mL of the simulated vaginal fluid and 0.5 mL of deionized water to obtain working solutions at several concentration levels. The calibration curves were prepared at 10 different concentrations of drug solutions and were linear over a concentration range from 0.709 to 11.390 μg/mL for clotrimazole and from 0.0709 to 1.1390 μL/mL for nifuratel with a correlation coefficient of >0.995.

### 2.13 Lap Shear Adhesion Test (Bioadhesion Test)

To assess the adhesion ability of a PBGE-PGGE-based hydrogel to fresh porcine skin incubated in a simulated vaginal fluid for 30 min, two identical skin stripes (25 (*L*) × 10 (*W*)) with a junction area of 1 cm^2^ using 0.020 mg of the hydrogel sample were used. The lap joint was compressed under a 300 g weight for 30 min. The two ends of the skin stripes were then mechanically clamped in a solid sample fixture of a rheometer (Thermoscientific HAAKE MARS 40) and stretched at a speed of 1 mm min^–1^ until separation. The tensile force–extension curves were recorded and analyzed. The adhesion strength was calculated by the maximum load divided by the initial bonded area. Each sample was tested a minimum of three times and averaged.

### 2.14 Cell Culture

Dermal microvascular endothelium cells (HMEC-1) and human cervical cancer endothelial (HeLa) cells were grown according to the procedure described previously.^36^

### 2.15 Determination of Cytotoxicity

The effects of the star-shaped copolyethers (PBGE-PGGE_1, PBGE-PGGE_2, PBGE-PGGE_3), star-shaped copolyethers carrying different amounts of clotrimazole (PBGE-PGGE_1_CLOT_a–d, PBGE-PGGE_2_CLOT_a–c, and PBGE-PGGE_3_CLOT_a–d), star-shaped copolyethers loaded with different amounts of nifuratel (PBGE-PGGE_1_NIF_a–e, PBGE-PGGE_2_NIF_a–f, and PBGE-PGGE_3_NIF_a–b), and free drugs (clotrimazole and nifuratel, respectively) on the cell viability were determined using the (3-[4,5-dimethylthiazol-2-yl]-2,5 diphenyl tetrazolium bromide) (MTT) assay.

To the 96-well plates containing cells at a density of 1.5 × 10^4^ cells/well in an appropriate medium, different concentrations of all compounds were added. Then, cells were incubated with the star-shaped copolyethers for 24 and 48 h in a 37 °C humidified atmosphere containing 5% CO_2_. After the incubation time, cells were washed with 50 μL of phosphate-buffered saline (PBS). The cells were then incubated for 3 h under normal culture conditions after the addition of 50 μL of a 0.5 mg/mL MTT solution in PBS to each well. After incubation, the remaining MTT solution was removed, and the resulting formazan precipitate was dissolved in DMSO (100 μL/well). The conversion of the tetrazolium salt (MTT) to a colored formazan by mitochondrial and cytosolic dehydrogenases is a marker of cell viability. Prior to absorbance measurement, plates were vortexed for 1 min, and the absorbance at 570 nm was measured using a PowerWave HT Microplate Spectrophotometer (BioTek).

### 2.16 Coefficient of Drug Interaction (CDI)

Compounds may act in a synergistic manner, which means that their combined effect is greater than the sum of the effects of their individual actions. The MTT method allows the calculation of a coefficient drug interaction (CDI) for their various combinations. The value of the CDI coefficient is defined by the formula:
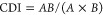
1where *AB* is the absorbance ratio of cells treated with a combination of two compounds relative to the control and *A* and *B* are the absorbance ratios of cells treated with a single compound relative to the control.

As in the case of the MTT assay described above, the control consisted of untreated cells.

A CDI value <1 shows synergism, CDI < 0.7 shows significant synergism, CDI = 1 shows additivity, and CDI > 1 shows antagonism.^38^ PBGE-PGGE_1_CLOT_d and PBGE-PGGE_1_NIF_e in the first variant and PBGE-PGGE_2_CLOT_c and PBGE-PGGE_2_NIF_e in the second variant were added to the cells seeded as described in the MTT method above separately and in a mixture containing varying levels of clotrimazole and nifuratel but at a final concentration range of 0.1–200 μM. Cells were incubated with the compounds for 24 and 48 h in a 37 °C humidified atmosphere containing 5% CO_2_. The assay was then performed according to the described MTT procedure, and the results were substituted into the formula.

### 2.17 Microscopic Images

Dermal microvascular endothelium cells (HMEC-1) and human cervical cancer endothelial cells (HeLa) were suspended in a culture medium at 500,000 and 400,000 cells/mL, respectively, and seeded outside the cell culture insert (ibidi, Munich, Germany; www.ibidi.de). After 24 h (after ensuring that the cells covered the entire free surface outside the inset), 30 μL volume of the respective gel (pure gel (PBGE-PGGE_1), gel with clotrimazol (PBGE-PGGE_1_CLOT_d), gel with nifuratel (PBGE-PGGE_1_NIF_e), gel containing equal parts of both drugs (PBGE-PGGE_1_CLOT/NIF), pure gel (PBGE-PGGE_2), gel with clotrimazol (PBGE-PGGE_2_CLOT_c), gel with nifuratel (PBGE-PGGE_2_NIF_e), and gel containing equal parts of both drugs (PBGE-PGGE_2_CLOT/NIF)) was pipetted into each chamber of the ibidi insert. The ibidi inserts were removed, and images were taken immediately and after 24 h incubation with gels, at 4× magnification, using a Nikon ECLIPSE E200 microscope and ImageJ software (NIH, Rockville).

### 2.18 Gene Expression

The expression level of NF-κB marker gene *NFKBIA* and cytokine genes *IL1* and *TNFa* was determined by quantitative real-time reverse transcription-polymerase chain reaction (RT-PCR). Aliquots of 1.5 × 10^6^ HeLa and HMEC-1 cells were cultured for 15, 30, 60, 90, 120, and 150 min with a clotrimazole or nifuratel concentration of 10 μM to select the most appropriate incubation time with drugs. After incubation, cells were washed once with PBS, and total cellular RNA was isolated using a TRI reagent (Sigma-Aldrich) according to the manufacturer’s protocol. Complementary DNA (cDNA) was transcribed from mRNA using a high-capacity cDNA reverse transcription kit (ThermoFisher) and used for real-time PCR amplification with a GoTaq qPCR Master Mix (Promega) according to the manufacturer’s protocol. Each 16 μL reaction volume contained 1 μL of cDNA and 0.25 μM forward and reverse intron-spanning primers (for primer sequences, see Table 1). The reference genes (HPRT1 and TBP) were selected according to the GeNorm procedure. The sequences of primers are presented in Table 2. PCR reactions were performed in 96-well microplates using the CFX96 Real-Time PCR Detection System (Bio-Rad). The expression level of assayed genes was expressed as the number of cognate mRNA copies per 1000 copies of geometric-averaged mRNA for reference genes.

**Table 1:** Primer Sequences

gene	forward and reverse sequences (5′–3′)
*HPRT1*	Fw: TGACACTGGCAAAACAATGCA
	Rv: GGTCCTTTTCACCAGCAAGCT
*TBP*	Fw: CACGAACCACGGCACTGATT
	Rv: TTTTCTTGCTGCCAGTCTGGAC
*NFKBIA*	Fw: TGAAGGCTACCAACTACAATGGC
	Rv: TGACATCAGCACCCAAGGACAC
*IL1*	Fw: GGACAGGATATGGAGCACAAGTG
	Rv: ACACGCAGGACAGGTACAGATTC
*TNFa*	Fw: TCCCCAGGGACCTCTCTCTA
	Rv: GAGGGTTTGCTACAACATGGG

**Table 2:** Characteristics of Synthesized Polyether-Based Unimolecular Micelles Constructed of 3-*star*-[(Poly(benzyl glycidyl ether))-*block*-(poly(glyceryl glycerol ether))] (PBGE-PGGE)

copolymer	core (number of BGE units per one arm), 3× (^1^H NMR)	*M*_w_/*M*_n_ PBGE, (GPC, CH_2_Cl_2_)	shell (number of IGG units), ^1^H NMR	*M*_n_ PBGE-PGGE, (^1^H NMR)	*M*_n_/*M*_w_ (GPC, DMF)	*T*_g_, °C	*d*, nm (DLS, 25 °C)^a^	PDI (DLS, 25 °C)
PBGE-PGGE_1	15	1.12	28	20,000	1.10	–17.6	25	0.266
PBGE-PGGE_2			86	45,700	1.16	–14.6	30	0.248
PBGE-PGGE_3			120	60,800	1.17	–16.2	28	0.130

a DLS measurements were performed for the aqueous solutions of each copolymer at *C* = 10 mg/mL.

After selecting the optimal incubation time with pure drugs, aliquots of 1.5 × 10^6^ of HeLa and HMEC-1 cells were cultured for 2 h with the star-shaped copolyethers (PBGE-PGGE_1, PBGE-PGGE_2, PBGE-PGGE_3); PBGE-PGGE_1_CLOT_a–d, PBGE-PGGE_2_CLOT_a–c, and PBGE-PGGE_3_CLOT_a–d; star-shaped copolyethers carrying different amounts of clotrimazole (PBGE-PGGE_1_NIF_a–e, PBGE-PGGE_2_NIF_a–f and PBGE-PGGE_3_NIF_a–b); and star-shaped copolyethers carrying different amounts of nifuratel at a final drug (clotrimazole or nifuratel) concentration of 10 μM. Then, using a similar procedure, total cellular RNA was isolated, transcribed from mRNA to cDNA, and amplified to perform the PCR reaction. The expression level of assayed genes was expressed as the number of cognate mRNA copies per 1000 copies of geometric-averaged mRNA for reference genes.

### 2.19 Hemolysis

Human blood from healthy adult donors was obtained from a local blood bank. Blood was centrifuged for 10 min at 400 g to remove serum and buffy coat. Then, erythrocytes were washed four times with ten volumes of PBS buffer (pH = 7.4), followed by centrifugation for 10 min at 400 g. Erythrocytes were suspended in PBS buffer, the hematocrit was measured, and the erythrocyte suspension was diluted to the hematocrit of 2%. The erythrocyte suspension was mixed with PBGE-PGGE_1_CLOT_a–d, PBGE-PGGE_2_CLOT_a–c, and PBGE-PGGE_3_CLOT_a–d; star-shaped copolyethers carrying different amounts of clotrimazole (PBGE-PGGE_1_NIF_a–e, PBGE-PGGE_2_NIF_a–f, and PBGE-PGGE_3_NIF_a–b); and star-shaped copolyethers carrying different amounts of nifuratel in the same buffer to obtain a final hematocrit of 1% and drug (clotrimazole or nifuratel) concentration in the range between 0.1 and 100 μM. Samples were incubated for 24 or 48 h at 37 °C. Next, samples were centrifuged at 400 g for 10 min. The supernatant was removed and the absorbance of the supernatant at 540 nm was measured. For positive and negative control, erythrocyte suspensions in distilled water and in PBS were used, respectively. The hemolysis amount was calculated from the equation:
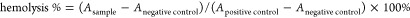
2For comparison, the experiment was performed for unloaded star-shaped copolyethers (PBGE-PGGE_1, PBGE-PGGE_2, and PBGE-PGGE_3) and neat drugs: clotrimazole and nifuratel.

### 2.20 Data Analysis

Statistical analysis was performed using StatSoft Statistica (version 13.1). Analysis of variance (ANOVA) with the Tukey post hoc test was used for result comparison. The results were considered significant at **p ≤ 0.01 and ***p ≤ 0.001.

## 3 Results and Discussion

### 3.1 Synthesis of Star-Shaped Poly(benzyl glycidyl ether)-*block*-poly(glyceryl glycerol ether) (3-*star*-[Poly(benzyl glycidyl ether)-*block*-poly(glyceryl glycerol ether)])

**Scheme 1:**
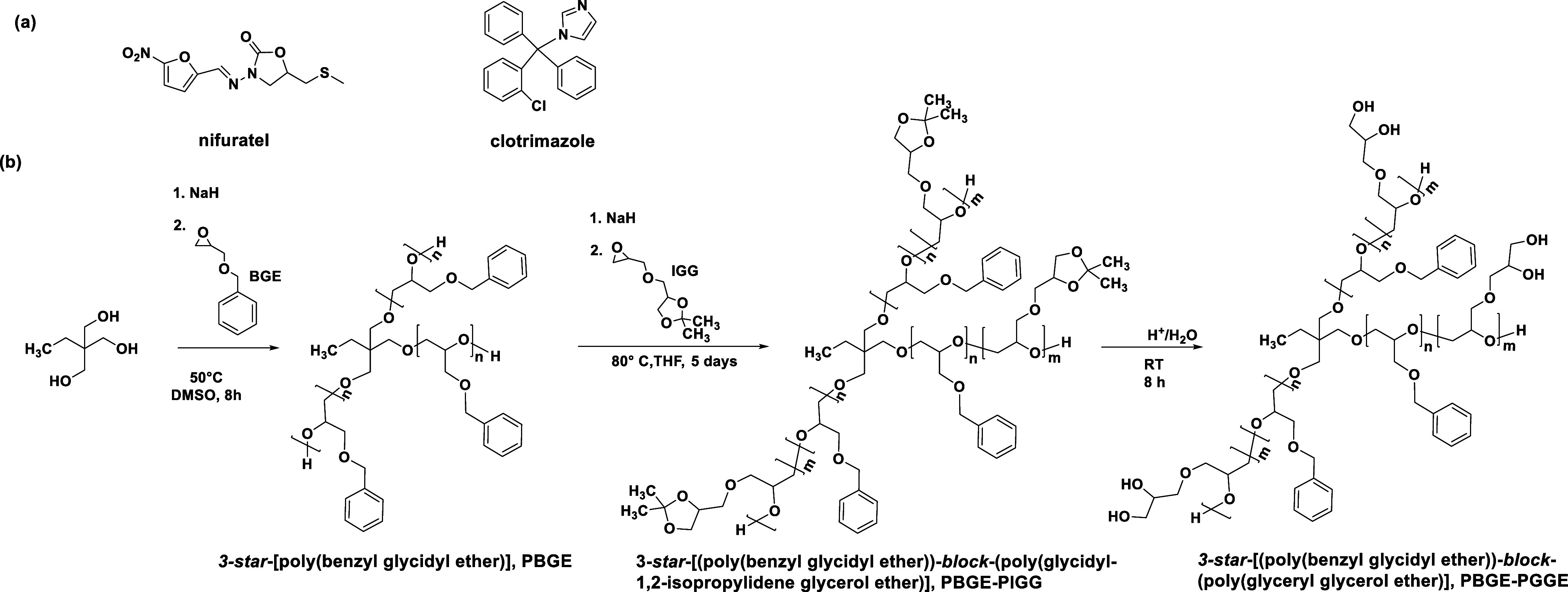
Chemical Structure of Investigated Hydrophobic Drugs (a) and the Synthetic Route of Copolyether-Based Star-Shaped Unimolecular Micelles (b) Applied for the Encapsulation of Drugs

For the encapsulation of poorly water-soluble aromatic ring-containing drugs such as nifuratel and clotrimazole (Scheme 1a), we synthesized three-arm unimolecular micelles with a distinctive aryl-enriched core composed of poly(benzyl glycidyl ether) and hydrophilic poly(glyceryl glycerol ether)-based shell (PBGE-PGGE) (Scheme 1b).

A poly(benzyl glycidyl ether) (PBGE)-based three-arm core consisted of 15 constitutional units per arm. The average length of each arm was determined based on ^1^H NMR spectra recorded in DMSO-*d*_6_ (Figure S1), comparing the integration of protons of the initiator’s CH_3_ groups (δ in the range from 0.80 to 0.86 ppm) and the integration of protons derived from aryl groups (δ at 7.21 ppm). In addition, molecular mass distribution was evaluated based on GPC in methylene chloride (*M*_w_/*M*_n_ = 1.12), as shown in Table 2. The hydrophobic PBGE chains were extended by anionic copolymerization of isopropylidene glycidyl ether, yielding a hydrophilic segment precursor per arm. DP*_n_* of poly(isopropylidene glycidyl ether) (PIGG) was estimated by comparing the integration of protons of CH_3_ groups of isopropylidene moieties present in the shell (δ at 1.31 and 1.26 ppm) and benzyl protons coming from the core (Figures S2, S4 and S6). Three copolymers differing in the PIGE length, i.e., 28, 86, and 120 repeating units, respectively, per arm, were obtained. Acidification of the synthesized copolymers resulted in the formation of a hydrophilic shell with 1,2-diols, yielding a series of 3-*star*-[(poly(benzyl glycidyl ether))-*block*-(poly(glyceryl glycerol ether))] (PBGE-PGGE) copolymers with a low-molecular-weight dispersity index, which was determined by GPC using DMF as an eluent (Table 2). In addition, ^1^H DOSY NMR spectra confirmed that the hydrophobic core of each copolymer was covalently bound with the hydrophilic shell (Figures S8–S10), as the diffusion coefficient of benzyl protons was comparable with the diffusion coefficient of GGE repeating units.

### 3.2 Encapsulation of Aromatic Group-Containing Drugs in the PBGE-PGGE Structure

PBGE-PGGE unimolecular micelles with an aryl-enriched core were applied to encapsulate hydrophobic drugs bearing aromatic groups, namely, clotrimazole and nifuratel. Clotrimazole, a poorly water-soluble drug in whose structure nonconjugated three aryl rings and an imidazole moiety can be distinguished, is routinely applied in the treatment of candidiasis. Nifuratel, a nitrofuran derivative, is used to treat vaginosis caused by fungi, bacteria, and protozoans. The low solubility of both clotrimazole and nifuratel in in the aqueous media limits their bioavailability. The process of the solubilization of investigated drugs was carried out according to the ultrasound-assisted solvent evaporation method using methanol. Then, the drug–copolymer mixture was dissolved in the deionized water and filtrated to remove the nonencapsulated drug. Both drug loading capacity and encapsulation efficiency of both drugs are given in Table 3. Based on the drug encapsulation data, it was found that, contrary to nifuratel, clotrimazole displayed higher affinity to the PBGE-based hydrophobic core. The lack of the evident effect of the length of the hydrophilic segment of the macromolecule on the encapsulation efficiency of clotrimazole was observed (Table 3). It is worth noting that in the case of nifuratel, the usage of the higher amount of the drug for the encapsulation process resulted in a diminished amount of the encapsulated drug. This behavior indicates that nifuratel does not interact strongly with the poly(benzyl glycidyl ether) segment and can only be surface-bound. Then, upon suspending nifuratel-loaded unimolecular micelles in water, drug molecules can easily precipitate. This behavior was more evident for the PBGE-PGGE_1 copolymer whose shell was the shortest among all investigated copolymers, i.e., DP*_n_* shell/DP*_n_* core is equal to approximately 1.87.

**Table 3:** Formulations of Nifuratel and Clotrimazole Encapsulated within 3-*star*-[(Poly(benzyl glycidyl ether))-*block*-(poly(glyceryl glycerol ether))] Unimolecular Micelles Differing in the Drug Loading Capacity^a^

formulation	drug loading, mg/g^b^	encapsulation efficiency, %^c^
Nifuratel-Loaded Copolymers		
PBGE-PGGE_1_NIF_a	39.1	14
PBGE-PGGE_1_NIF_b	46.4	8
PBGE-PGGE_1_NIF_c	58.2	14
PBGE-PGGE_1_NIF_d	80.0	59
PBGE-PGGE_1_NIF_e	129.7	90
PBGE-PGGE_2_NIF_a	21.3	42
PBGE-PGGE_2_NIF_b	37.0	59
PBGE-PGGE_2_NIF_c	52.0	51
PBGE-PGGE_2_NIF_d	142.9	94
PBGE-PGGE_2_NIF_e	245.1	100
PBGE-PGGE_3_NIF_a	22.5	100
PBGE-PGGE_3_NIF_b	44.9	96
Clotrimazole-Loaded Copolymers		
PBGE-PGGE_1_CLOT_a	51.0	59
PBGE-PGGE_1_CLOT_b	172.6	100
PBGE-PGGE_1_CLOT_c	189.8	100
PBGE-PGGE_1_CLOT_d	338.8	98
PBGE-PGGE_2_CLOT_a	18.4	81
PBGE-PGGE_2_CLOT_b	43.0	100
PBGE-PGGE_2_CLOT_c	175.4	100
PBGE-PGGE_3_CLOT_a	17.8	100
PBGE-PGGE_3_CLOT_b	28.65	100
PBGE-PGGE_3_CLOT_c	47.1	100
PBGE-PGGE_3_CLOT_d	115.3	100

a Encapsulation efficiency of drugs in copolyethers is given for comparison.

b Drug loading capacity—the weight ratio of the drug to polymer in the formulation.

c Encapsulation efficiency—the ratio of the drug amount in micelles to the total amount of the drug applied for the preparation of formulation.

### 3.3 Study of the Release of Active Compounds from Drug-Loaded PBGE-PGGE Unimolecular Micelles

**Figure 1:**
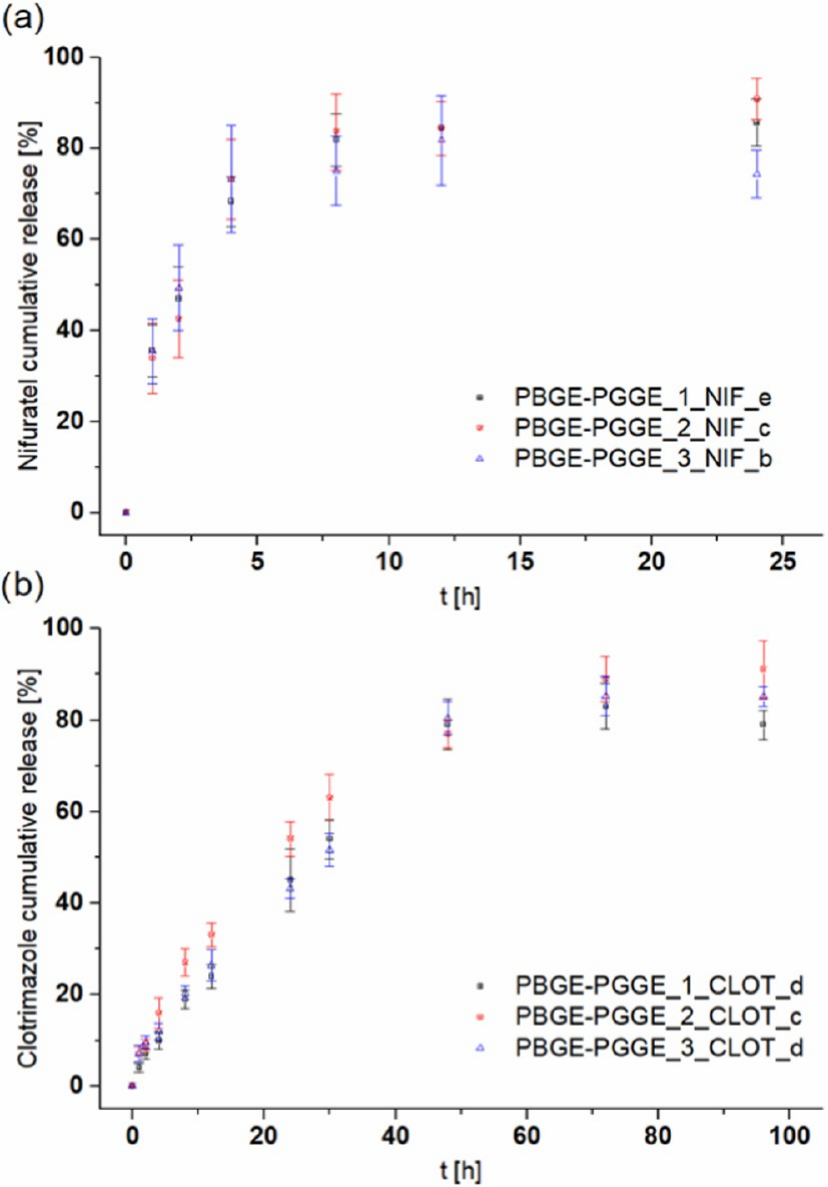
*In vitro* release profiles of nifuratel (a) and clotrimazole (b) from drug-loaded copolyether unimolecular micelles.

To evaluate the potential of PBGE-PGGE unimolecular micelles as drug delivery systems, it was necessary to verify whether the encapsulated drugs were able to diffuse gradually out of the structures. For this goal, *in vitro* drug release experiments for drug-loaded copolymers at the conditions corresponding to the vagina environment were performed. A drug release study was carried out on copolyether formulations that contain the same drug-to-macromolecule molar ratio, however, using copolymers differing in the length of the hydrophilic shell. Contrary to traditional micelles,^39^ the initial burst release of both nifuratel and clotrimazole was hardly observed for all star-shaped copolyethers investigated here (Figure 1). Experiments of drug release from unimolecular micelles also did not show the influence of the length of the hydrophilic shell (DP*_n_* in the range from 28 to 120) on the rate of drug release.

The lower affinity of the nifuratel to poly(benzyl glycidyl ether) core was also observed based on the drug release profiles. The complete removal of encapsulated nifuratel from PBGE-PGGE unimolecular micelles was observed after approximately 7 h and was four times faster than it was observed for clotrimazole (45 h). In general, no dependence in the rate of both nifuratel and clotrimazole release from PBGE-PGGE unimolecular micelles differing in the ratio of DP*_n_* (shell)/DP*_n_* (core) was observed.

### 3.4 Formation of Hydrogel Systems Based on PBGE-PGGE Star-Shaped Copolyethers

**Scheme 2:**
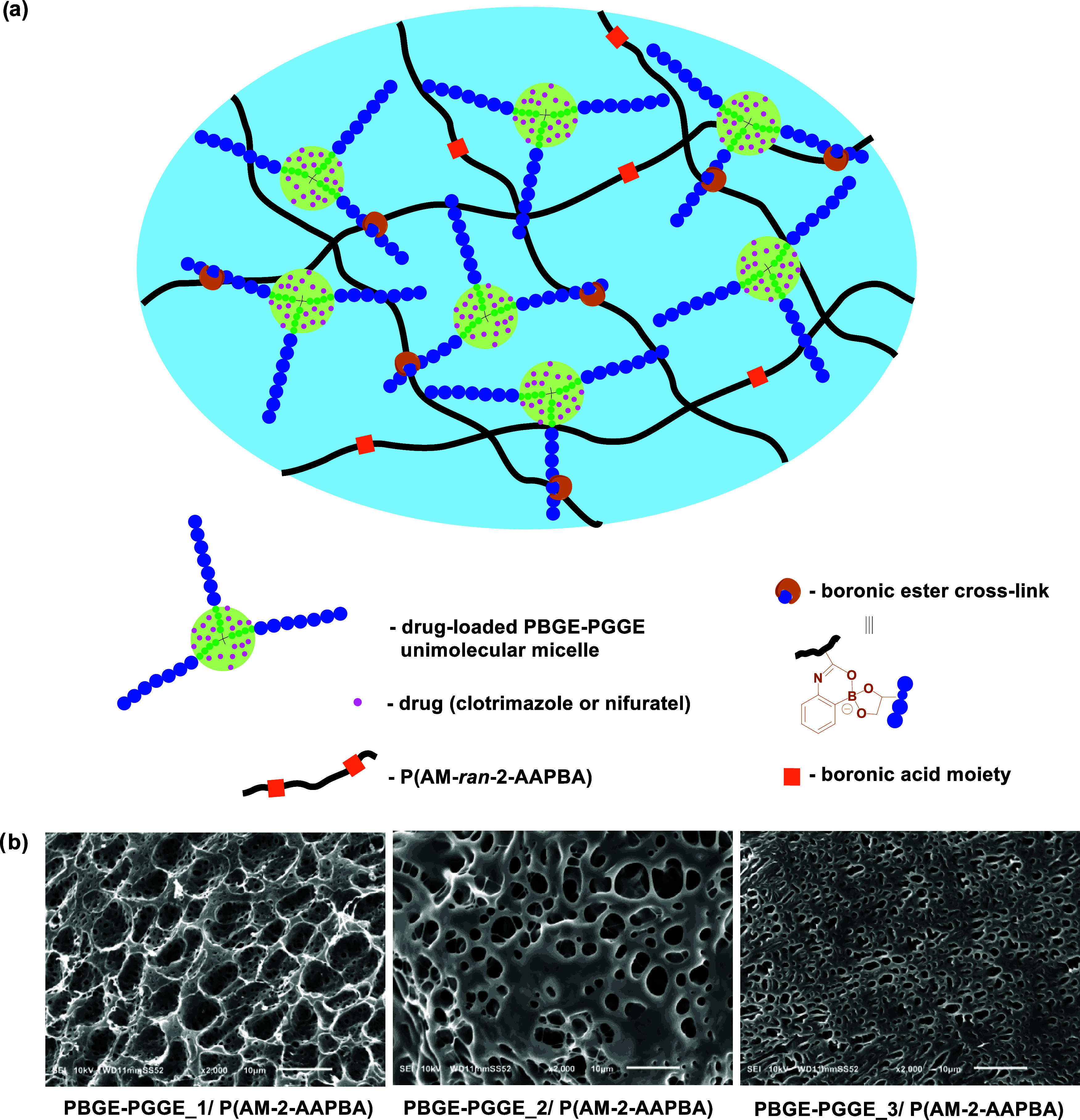
Schematic Illustrating the Network Construction Based on Cross-Linked PBGE-PGGE Unimolecular Micelles with P(AM-*ran*-2-AAPBA) via Reversible Boronic Esters (a) and SEM Images of Hydrogels Constructed of PBGE-PGGE Differing in the Length Ratio of the Shell and the Core (b)

The prolonged action of a drug in the afflicted area is crucial to ensure the efficiency of the anticancer therapy via the vaginal route. The use of a therapeutic formulation in the form of drug-loaded unimolecular micelles alone would lead to uncontrolled leakage from the vagina due to its inadequate rheological properties. The construction of a hydrogel composed of drug-loaded unimolecular micelles can ensure the maintenance of drugs in the afflicted area, avoiding their premature removal, which is also often observed in the case of commercially accessible standard suppositories. In view of the intravaginal therapy, the construction of the hydrogel should be based on reversible cross-links. This concept of hydrogel design can ensure the formation of a continuous layer on the covered surface and its removal after the fulfillment of the function. Such hydrogels are determined as dynamic as the network integrity is controlled by the equilibrium of the reaction between a product (cross-link) and substrates. To obtain dynamic hydrogels, 1,2-diol units of the PGGE shell of amphiphilic copolyethers were employed for cross-linking with 2-acrylamidephenylboronic acid incorporated randomly along the acrylamide copolymer P(AM-2-AAPBA) containing a 10 mol % fraction of 2-AAPBA units (Scheme 2a). A carbonyl oxygen atom present in the proximity of the boronic acid moiety in a 2-AAPBA unit ensures the intramolecular coordination with the boron atom, reducing its p*K*_a_ value, which causes that 2-AAPBA units can be cross-linked with diols at both acidic and neutral pH.^40^ This behavior of 2-AAPBA groups assures the formation of stable hydrogels at the pH of the vagina. SEM images recorded for hydrogels (17 wt %) obtained by cross-linking of PBGE-PGGE copolymers differing in the length ratio of the shell to core via intermolecular boronic ester formation revealed the porous structure of all networks (Scheme 2b). Due to the reversible character of boronic ester formation, its dissociation, followed by covalent bond reformation, can make the network dynamic.

### 3.5 Rheological Properties

**Figure 2:**
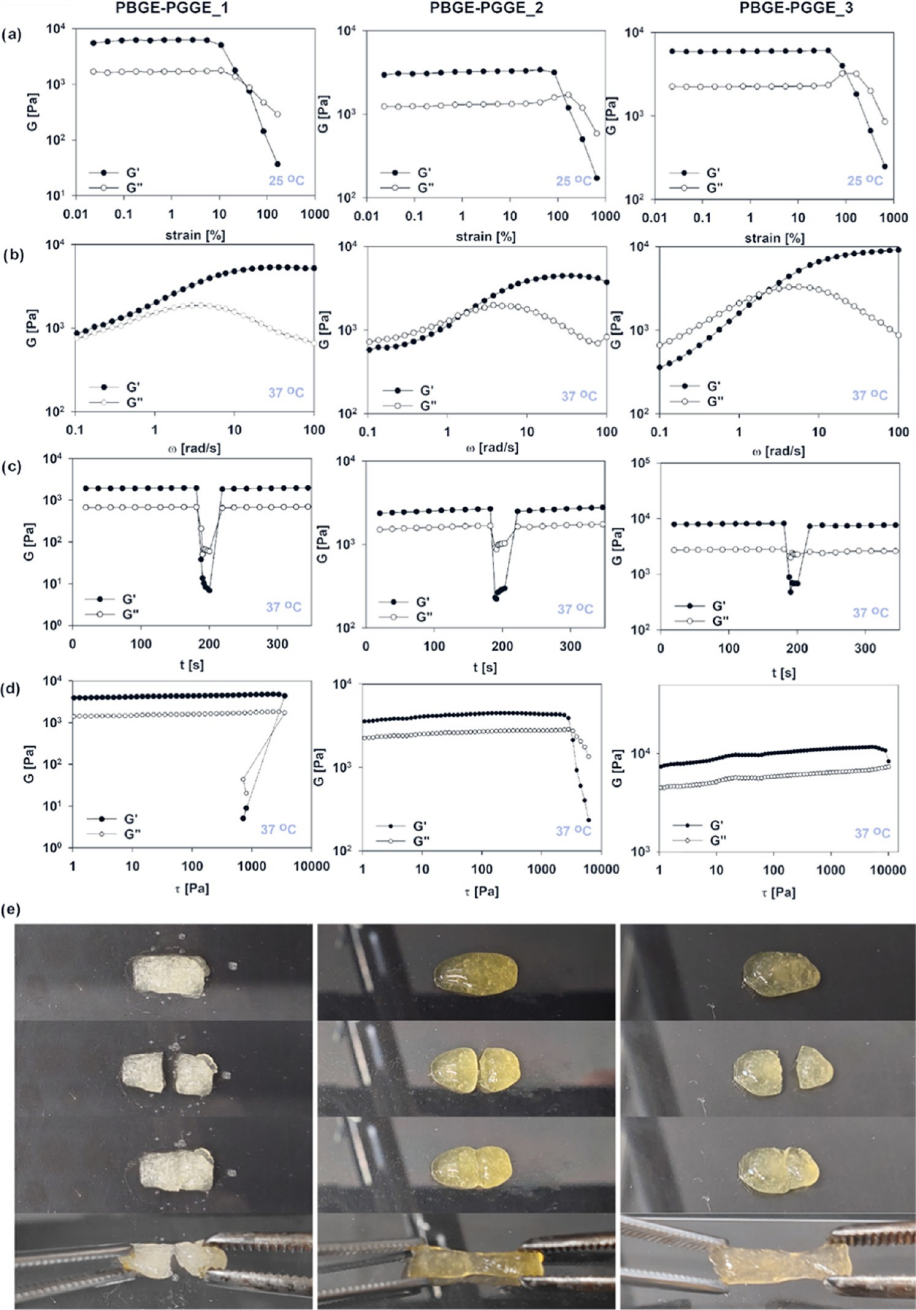
Strain sweep tests (a) and frequency sweep tests performed for hydrogel systems built of reversibly cross-linked PBGE-PGGE macromolecules differing in the hydrophilic shell length (b). Self-healing behavior of hydrogels (c) along with stress sweep experiments (d) performed at vagina conditions. The time-dependent self-healing process of hydrogels constructed of PBGE-PGGE macromolecules differing in the hydrophilic shell length at room temperature along with the visual demonstration of the ability of the hydrogel to reform from two distinct pieces (e). The fracture gradually vanished only in the case of hydrogels constructed of PBGE-PGGE_2 and PBGE-PGGE_2 macromolecules, leading to its complete decay.

A rheological study of hydrogels was carried out at room and physiological temperature to investigate the behavior of hydrogels at the administration stage and *in vivo* conditions (Figure 2), however the sysnthesized hydrogels displayed the structural stability in the investigated temperature range from 12 to 40 °C (Figure S11). The frequency sweep experiments performed for hydrogels constructed of PBGE-PGGE_2 and PBGE-PGGE_3 copolymers revealed the viscoelastic character of hydrogel networks in water (Figure 2b). In the higher frequency range, i.e., on the shorter time scales, the storage modulus (*G*′) exceeded the value of the loss modulus (*G*″). This is due to the fact that the lifetime of boronic ester cross-links was longer compared to the strain rate used. Reduction of the frequency resulted in the inversion of storage and loss moduli at the crossover frequency (ω_c_), which corresponds to the gelation point (gel–liquid transition) as an effect of the reshuffling of the cross-links. ω_c_ denotes the onset of macroscopic chain displacement, while at frequencies below ω_c_, i.e., longer time scales, the material starts to flow (*G*′ < *G*″) due to the dominant contribution of liquid behavior. The crossover frequency for both PBGE-PGGE_2 and PBGE-PGGE_3-based hydrogels was similar, at approximately 1.00 and 2.00 rad/s at 25 and 37 °C, respectively, indicating that differences in the shell length at the investigated range of used copolymers have a minor effect on the macromolecules’ dynamics in the dynamically cross-linked hydrogels. Contrary to hydrogels constructed of PBGE-PGGE_2 and PBGE-PGGE_3, hydrogels constructed of the PBGE-PGGE_1 copolymer displayed a predominant solid behavior in the investigated frequency range (from 0.1 to 100 rad/s). In the low-frequency range, the storage modulus decreased, however still, *G*′> *G*″. This behavior was observed at both 25 and 37 °C; however, the difference between *G*′ and *G*″ was lower at a higher temperature.

Based on the frequency sweep experiment performed for both neat hydrogel based on PBGE-PGGE_2 and its drug-loaded counterpart, no evident influence of drugs on the rheological properties of prepared networks was observed (Figure S12), which input that drugs at investigated concentrations do not modulate the structural properties of the network.

Strain sweep tests performed for hydrogels at 25 °C revealed the differences in the structural stability of the hydrogel networks constructed of star-shaped amphiphiles differing in the shell length (Figure 2a). At the low amplitude range, both *G*′ and *G*″ moduli exhibited a plateau, characteristic of the linear viscoelastic region, followed by a decrease in both moduli at an amplitude that is specific for each hydrogel. After exceeding the critical strain, at which the network is disrupted, the inversion of *G*′ and *G*″ was observed, and *G*″ became larger than *G*′. It is worth noting that in the case of a PBGE-PGGE_1-based hydrogel, only 10% of applied strain was necessary to disrupt the network at 25 °C, whereas, for networks based on PBGE-PGGE_2 and PBGE-PGGE_3 copolymers, this value ranged from 94 to 120%. This behavior input that besides boronic ester-based cross-links, the hydrophobic association between individual macromolecules occurs, even though aggregation of macromolecules in the aqueous solution at the concentration applied for the network construction was not observed.

An important feature of PBGE-PGGE-based hydrogels at body temperature is their self-healing properties. The experiments revealing the hydrogel’s response to the drastic applied strain were carried out at 37 °C (Figure 2c). When the strain was increased to γ = 300%, a significant decrease in both moduli was observed along with the inversion of *G*′ and *G*″, indicating the disruption of the network. Subsequent reduction of strain to 1% resulted in rapid regeneration of the hydrogel structure after strain-induced failure, as evidenced by the return of moduli to their original values (Figure 2c). The experiment confirmed that the structure of all investigated hydrogels can be recovered as a result of the fast reshuffling of the cross-links. Due to the dynamic character of the boronic ester cross-links, hydrogels composed of star-shaped copolyethers can be easily shaped and form a continuous layer on the covered surface. This characteristic is highly important in view of biomedical applications because it facilitates hydrogel deposition at the target site and ensures proper contact with the tissue, providing control over local drug delivery.

The self-healing experiments performed for hydrogels, however, at room temperature by simply cutting a piece of each gel into two pieces, revealed the difference in the reassembling ability into one piece (Figure 2e). Both PBGE-PGGE_2 and PBGE-PGGE_3-based hydrogels were self-healable at room temperature after approximately 15 min, whereas the hydrogel platform based on PBGE-PGGE_1 was not repairable at this time (Figure 2e). This behavior indicates the contribution of hydrophobic interactions in the network formed with a star-shaped PBGE-PGGE copolymer in which the shell is the shortest, i.e., the ratio of the DP*_n_*’s shell to core is the lowest (∼2). Since for the aqueous solution of PBGE-PGGE_1 at the concentration used in the hydrogel formulation, no aggregation effects were observed, it can be assured that the cross-linking process triggers the hydrophobic association formation.

Considering the possibility of the uncontrolled hydrogel drug carriers flowing out of the vagina, we investigated their yield points by performing the stress sweep experiment at 37 °C. The yield point is determined based on the crossover of both moduli, which is related to a transition from a solid to a liquid state at which the material displays a viscous flow. No yield stress was observed for the PBGE-PGGE_1 hydrogel (Figure 2d). In the case of PBGE-PGGE_2 and PBGE-PGGE_3 hydrogels, the flow of the samples was observed at high-stress values and was equal to 2.8 and 8.95 kPa, respectively (Figure 2d). Although all of the hydrogels were injectable (see video files), in the case of the hydrogel based on PBGE-PGGE_3, distinct resistance was observed during its injecting from the syringe, which limits its potential for intravaginal application.

### 3.6 Influence of the Star-Shaped PBGE-PGGE Copolyethers and Drug-Loaded Constructs on Cell Viability

**Figure 3:**
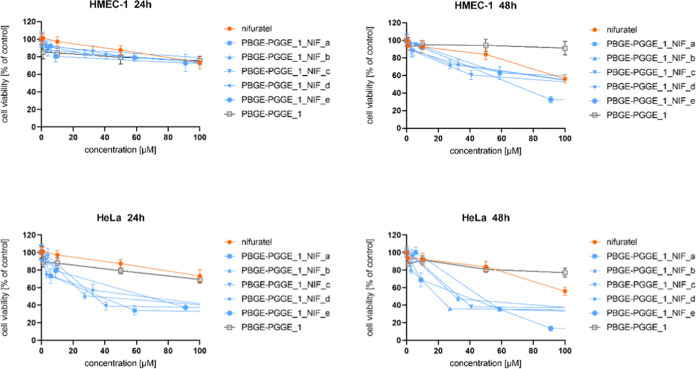
Influence of PBGE-PGGE_1_NIF_a–e on HMEC-1 (upper panel) and HeLa (bottom panel) cell viability after 24 and 48 h of incubation. The results are presented as the mean ± SD (*n* = 16).

**Figure 4:**
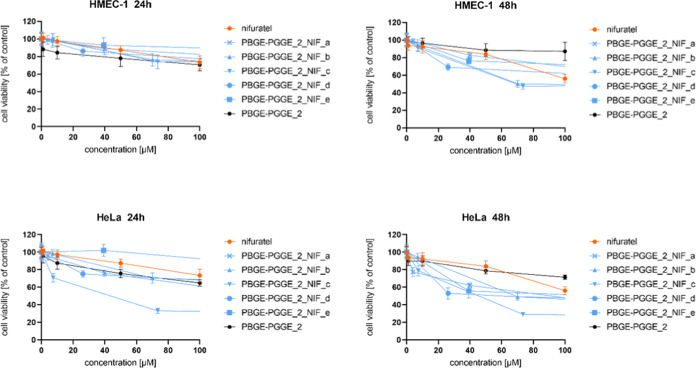
Influence of PBGE-PGGE_2_NIF_a–f on HMEC-1 (upper panel) and HeLa (bottom panel) cell viability after 24 and 48 h of incubation. The results are presented as the mean ± SD (*n* = 16).

**Figure 5:**
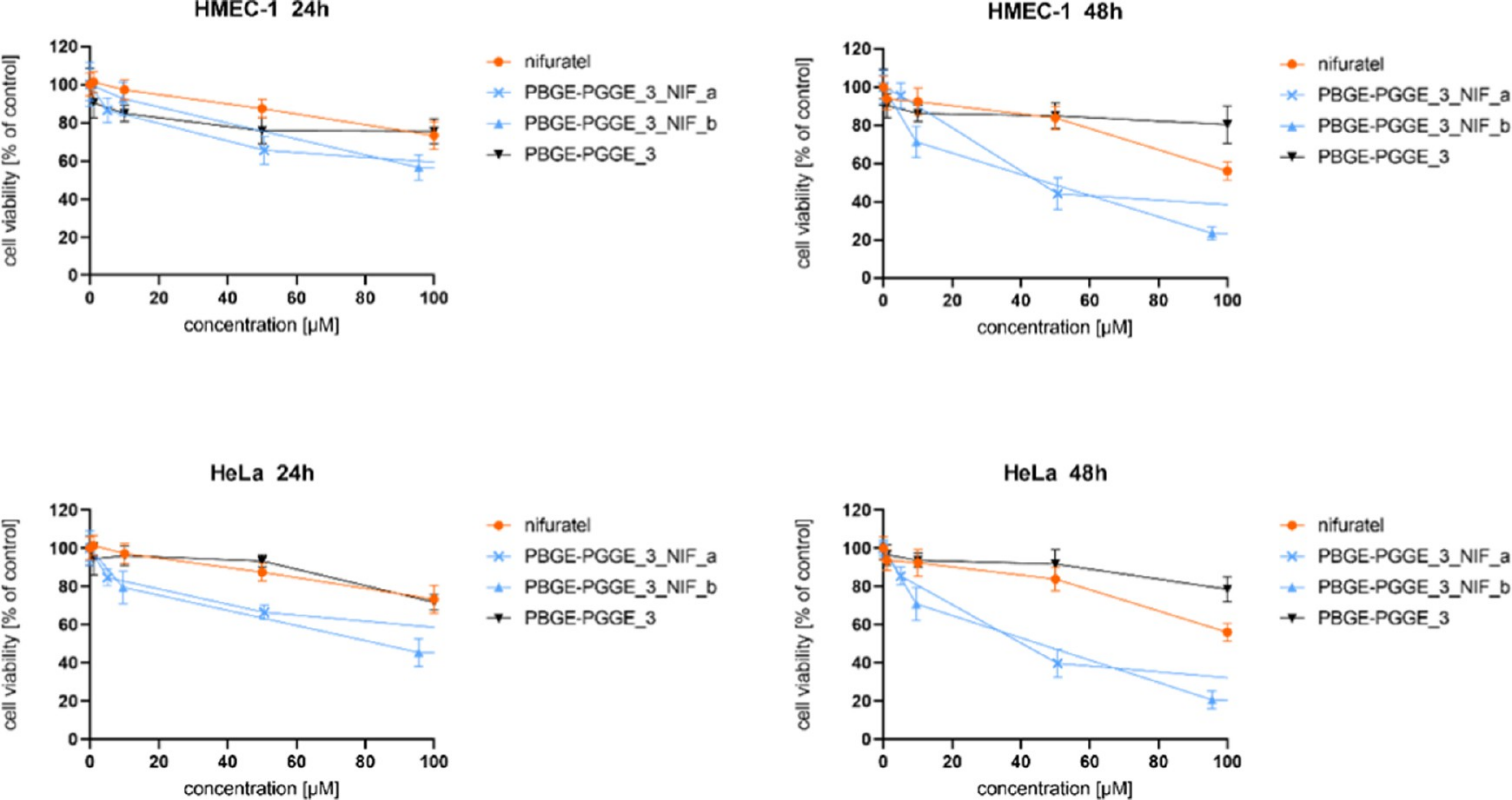
Influence of PBGE-PGGE_3_NIF_a–b on HMEC-1 (upper panel) and HeLa (bottom panel) cell viability after 24 and 48 h of incubation. The results are presented as the mean ± SD (*n* = 16).

**Figure 6:**
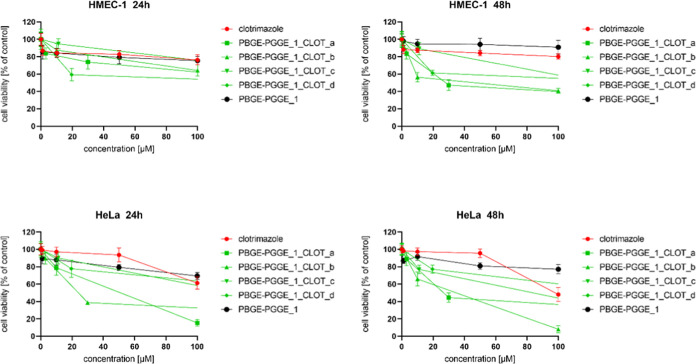
Influence of PBGE-PGGE_1_CLOT_a–d on HMEC-1 (upper panel) and HeLa (bottom panel) cell viability after 24 and 48 h of incubation. The results are presented as the mean ± SD (*n* = 16).

**Figure 7:**
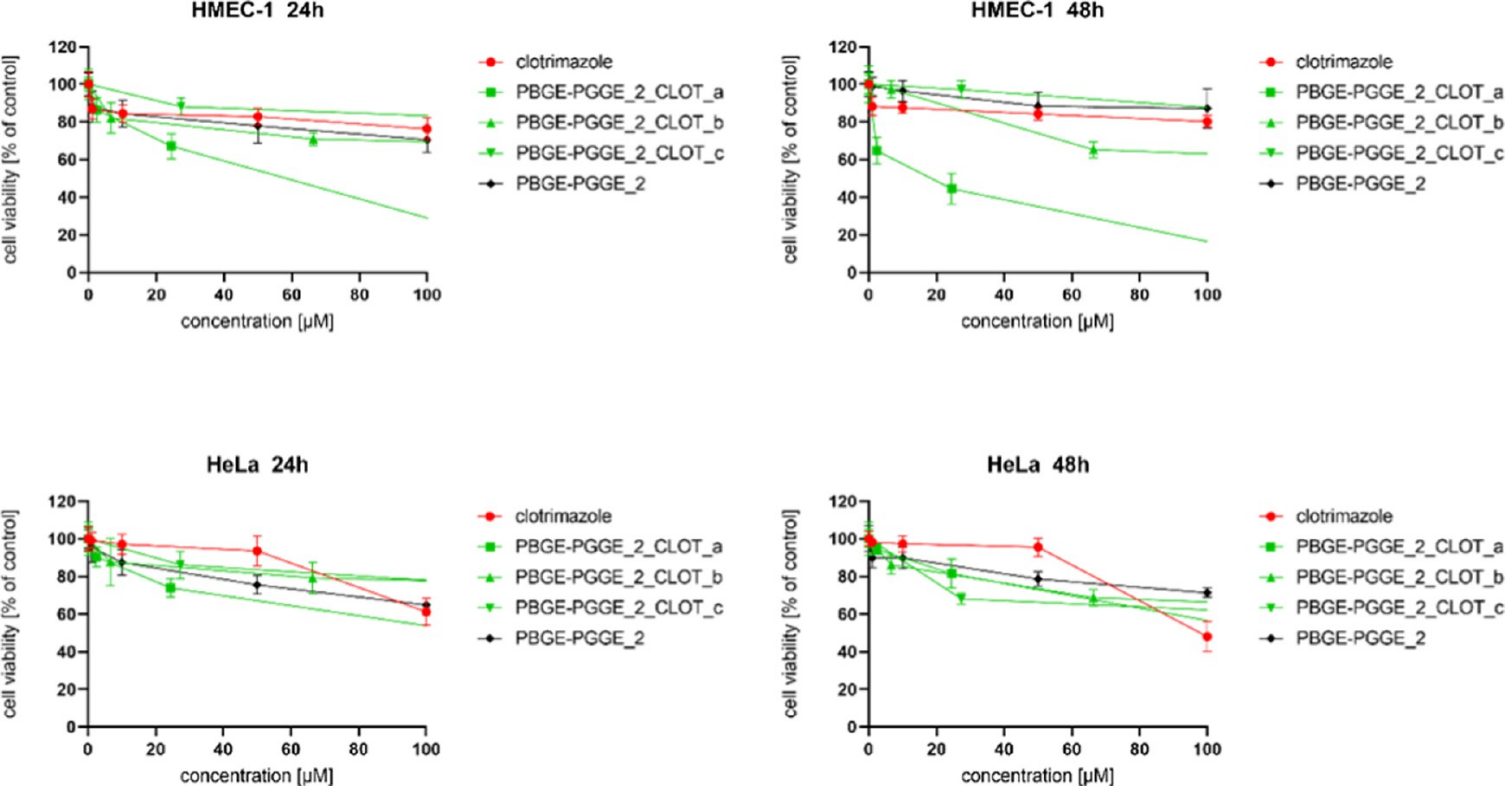
Influence of PBGE-PGGE_2_CLOT_a–c on HMEC-1 (upper panel) and HeLa (bottom panel) cell viability after 24 and 48 h of incubation. The results are presented as the mean ± SD (*n* = 16).

**Figure 8:**
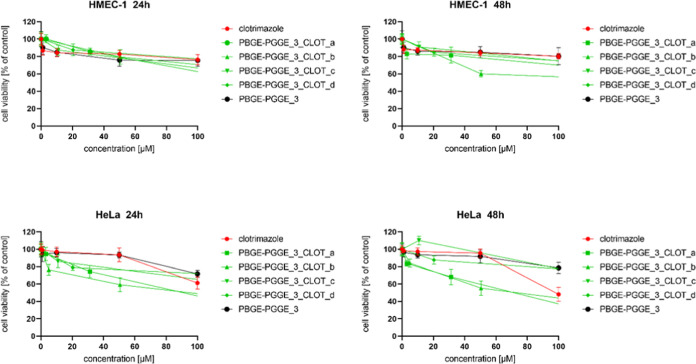
Influence of PBGE-PGGE_3_CLOT_a–d on HMEC-1 (upper panel) and HeLa (bottom panel) cell viability after 24 and 48 h of incubation. The results are presented as the mean ± SD (*n* = 16).

PBGE-PGGE copolyethers differing in the drug loading of both nifuratel and clotrimazole (Table 3) were prepared to determine the most effective formulation against cancer cells along with the most harmless action toward normal cells. The bioassays aimed to answer the question of whether PBGE-PGGE copolymers could effectively transport both drugs. Since the selected drugs differ in their mechanism of action on cancer cells, it was indispensable to verify whether there would be a synergistic effect of the two drugs by mixing unimolecular micelles loaded with nifuratel and clotrimazole, respectively. MTT experiments were performed by incubation of each drug formulation with cervical cancer cells (HeLa) and normal skin microvascular endothelial cells (HMEC-1) for 24 and 48 h, respectively. The cell viability curves for the drug-loaded constructs of different amounts of drugs (Table 3) compared to the free drugs and neat copolymers are presented in Figures 3–8. Based on their course, the IC_50_ was determined, informing about the concentration for which only half of the cells are alive (Tables 4 and 5). To allow a direct comparison of systems containing different amounts of the encapsulated drug with a free drug, cell viability scores were normalized according to drug content.

**Table 4:** Comparison of the IC_50_ Value for Neat Nifuratel and Nifuratel-Encapsulated Star-Shaped PBGE-PGGE Copolyethers in HeLa and HMEC-1 Cell Lines^a^

	HeLa		HMEC-1	
compound [μM]	24 h	48 h	24 h	48 h
nifuratel	339.41 ± 6.62	103.09 ± 6.84	315.47 ± 5.97	116.11 ± 5.45
PBGE-PGGE_1_NIF_a	53.42 ± 2.50***	26.21 ± 2.88***	>500	132.77 ± 4.25
PBGE-PGGE_1_NIF_b	40.25 ± 3.93***	55.62 ± 2.88***	>500	116.12 ± 4.89
PBGE-PGGE_1_NIF_c	62.58 ± 6.91***	61.04 ± 2.48***	>500	110.61 ± 3.85**
PBGE-PGGE_1_NIF_d	34.71 ± 6.82***	67.65 ± 3.93***	>500	211.62 ± 5.00
PBGE-PGGE_1_NIF_e	6.91 ± 5.71***	20.34 ± 5.24***	>500	118.99 ± 2.90
PBGE-PGGE_2_NIF_a	118.00 ± 2.66***	43.56 ± 1.93***	>500	223.36 ± 3.96
PBGE-PGGE_2_NIF_b	372.47 ± 3.58	139.05 ± 4.07	>500	181.33 ± 3.99
PBGE-PGGE_2_NIF_c	19.03 ± 3.88***	21.47 ± 3.77***	>500	252.65 ± 3.58
PBGE-PGGE_2_NIF_d	>500	42.72 ± 5.67***	>500	328.48 ± 4.51
PBGE-PGGE_2_NIF_e	45.35 ± 1.81***	5.52 ± 4.85***	>500	56.85 ± 4.41***
PBGE-PGGE_2_NIF_f	>500	64.80 ± 4.79***	>500	>500
PBGE-PGGE_3_NIF_a	101.52 ± 2.90***	33.20 ± 5.97***	131.23 ± 4.95***	64.99 ± 5.42***
PBGE-PGGE_3_NIF_b	92.80 ± 7.88***	27.98 ± 6.70***	229.84 ± 5.15	68.17 ± 6.19***

a The IC_50_ values are presented as mean ± SD. The results were considered significant at **p ≤ 0.01 and ***p ≤ 0.001 with regard to neat nifuratel.

**Table 5:** Comparison of the IC_50_ (μM) Value for Neat Clotrimazole and Clotrimazole Encapsulated in Star-Shaped PBGE-PGGE Copolyethers in HeLa and HMEC-1 Cell Lines^a^

	HeLa		HMEC-1	
compound	24 h	48 h	24 h	48 h
clotrimazole	127.42 ± 7.11	97.33 ± 6.46	> 500	> 500
PBGE-PGGE_1_CLOT_a	32.74 ± 1.15***	38.40 ± 3.13***	228.10 ± 4.75***	49.95 ± 2.82***
PBGE-PGGE_1_CLOT_b	38.29 ± 6.19***	16.35 ± 6.03***	225.03 ± 4.45***	26.58 ± 5.44***
PBGE-PGGE_1_CLOT_c	>500	157.07 ± 3.16	>500	314.26 ± 3.08***
PBGE-PGGE_1_CLOT_d	>500	286.89 ± 3.27	389.22 ± 4.75***	166.07 ± 2.28***
PBGE-PGGE_2_CLOT_a	112.19 ± 2.84***	116.18 ± 5.17	32.42 ± 5.43***	9.00 ± 7.62***
PBGE-PGGE_2_CLOT_b	274.51 ± 6.47	115.21 ± 3.67	271.12 ± 4.60***	153.88 ± 5.83***
PBGE-PGGE_2_CLOT_c	>500	433.13 ± 2.60	>500	>500
PBGE-PGGE_3_CLOT_a	49.31 ± 4.53***	30.42 ± 6.02***	146.74 ± 4.26***	>500
PBGE-PGGE_3_CLOT_b	41.14 ± 7.17***	39.85 ± 6.55***	219.02 ± 5.20***	199.71 ± 4.55***
PBGE-PGGE_3_CLOT_c	>500	471.44 ± 3.46	>500	>500
PBGE-PGGE_3_CLOT_d	>500	>500	>500	>500

a The IC_50_ values are presented as mean ± SD. The results were considered significant at **p ≤ 0.01 and ***p ≤ 0.001 with regard to neat clotrimazole.

It is worth noting that PBGE-PGGE copolyethers, regardless of the ratio of DP*_n_* (shell)/DP*_n_* (core), were not cytotoxic toward both cervical cancer cells (HeLa) and normal skin microvascular endothelial cells (HMEC-1).

PBGE-PGGE constructs turned out to be excellent carriers of nifuratel. Comparing IC_50_ values for nifuratel formulations (Table 4), it can be concluded that nifuratel loaded in unimolecular micelles is more effective in comparison to free drugs. The cytotoxicity was not strictly correlated with the amount of the drug entrapped in the analyzed structures. The obtained results were, however, surprising in two aspects. First, the multiple increase in anticancer activity of some constructs with nifuratel, i.e., PBGE-PGGE_1_NIF_e or PBGE-PGGE_2_NIF_c, was extraordinary (Table 4). Second, the encapsulated nifuratel was not only more effective than the free drug, but what is truly unique, the use of the PBGE-PGGE_1 and PBGE-PGGE_2 polymers gave nifuratel selectivity against cancer cells. The selectivity of the encapsulated nifuratel is best shown by IC_50_ values of nifuratel determined for both HeLa and HMEC-1 cells (Table 4). This is a unique result, unprecedented in the literature. So far, Zheng et al. have shown that nifuratel can inhibit the proliferation and induced apoptosis of gastric cancer cells (cell lines SGC-7901 and BGC-823); however, they highlighted that it is essential to enhance the selectivity and reduce the toxicity effects of nifuratel on normal cells.^20^ As first, in this work, we demonstrate a carrier promoting the nifuratel selectivity providing drug formulations that selectively affect cervical cancer cells.

Although PBGE-PGGE constructs were suitable also to encapsulate clotrimazole (Table 3), the biological effect was, however, slightly worse in comparison to nifuratel (Table 4). Figures 6–8 present the viability curves for constructs loaded with different amounts of clotrimazole (PBGE-PGGE_1_CLOT_a–d, PBGE-PGGE_2_CLOT_a–c and PBGE-PGGE_3_CLOT_a–d, respectively) compared to the free drug. Analyzing the IC_50_ values contained in Table 4, it can be concluded that all systems (except for PBGE-PGGE_2_CLOT_a) retained the tumor cell selectivity characteristic for the free drug.^22,41^ The cytotoxicity of drug-loaded copolymers was not correlated with the amount of the drug entrapped in the analyzed structures. Comparing the clotrimazole loading within the copolymer (Table 3) with the IC_50_ values (Table 5), it can be concluded that systems with a low drug loading were the most effective, as previously observed for star-hyperbranched amphiphilic constructs,^36^ and this can be explained that the high clotrimazole content in the polymer structure hindered its release and immediate action.

Encouraged by the obtained results, we decided to check whether drugs encapsulated in the tested systems can act synergistically while maintaining reduced toxicity toward noncancer cells. Two PBGE-PGGE_1 and PBGE-PGGE_2 polymer systems containing 338.8 and 175.4 mg/g clotrimazole and 129.7 and 245.1 mg/g nifuratel, respectively, were selected for the study. Both pairs were selected so that the single constructs were characterized by low cytotoxicity against noncancerous HMEC-1 cells and, paradoxically, were not the most effective systems, which allowed us to observe the potential synergism of their action. Their ability to formulate injectable hydrogels, which would allow them to be used as a potential topical drug formulation in the treatment of cervical cancer, was significantly noticeable. The results of the combined administration of equal parts of the clotrimazole and nifuratel solutions in both PBGE-PGGE_1 and PBGE-PGGE_2 copolymers are presented in Table 6.

**Table 6:** Comparison of the IC_50_ (μM) Value for Clotrimazole and Nifuratel Encapsulated in Star-Shaped Amphiphiic Copolyethers in HeLa and HMEC-1 Cell Lines^a^

	HeLa		HMEC-1	
compound	24 h	48 h	24 h	48 h
PBGE-PGGE_1_CLOT_d	>500	286.89 ± 3.27	389.22 ± 4.75	166.07 ± 2.28
PBGE-PGGE_1_NIF_e	6.91 ± 5.71	20.34 ± 5.24	>500	118.99 ± 2.90
PBGE-PGGE_1_CLOT_d/NIF_e	65.27 ± 5.53***	45.58 ± 3.77***	>500	123.85 ± 1.63***
PBGE-PGGE_2_CLOT_c	>500	433.13 ± 2.60	>500	>500
PBGE-PGGE_2_NIF_e	>500	64.80 ± 4.79	>500	>500
PBGE-PGGE_2_CLOT_c/NIF_e	8.19 ± 3.37^***,+++^	1.11 ± 2.37^***,+++^	66.79 ± 1.49^***,+++^	26.59 ± 3.56^***,+++^

a The IC_50_ values are presented as mean ± SD. The results were considered significant at **p ≤ 0.01 and ***p ≤ 0.001 with regard to encapsulated clotrimazole and at ^++^*p* ≤ 0.01 and ^+++^*p* ≤ 0.001 with regard to encapsulated nifuratel.

Simultaneous administration of nifuratel and clotrimazole, encapsulated in the structure of star-shaped unimolecular micelles based on poly(benzyl glycidyl ether)-poly(glyceryl glyceryl ether), results in a significant synergism of action for both PBGE-PGGE_1_CLOT/NIF and PBGE-PGGE_2_CLOT/NIF systems (Table 6). Synergism is observed when the combined effect of two compounds is greater than the sum of the effects of their individual action. From the MTT results, a coefficient drug interaction (CDI), defined as the ratio of the absorbance ratio of cells treated with a combination of two compounds to the control and the ratio of absorbance of cells treated with a single compound versus the control, was determined. A CDI value <0.7 indicates a significant synergism.^38^ The absorbance value registered for different concentrations of PBGE-PGGE_1_CLOT_d and PBGE-PGGE_1_NIF_e and for PBGE-PGGE_2_CLOT_c and PBGE-PGGE_2_NIF_e, separate or combined, allowed to determine the coefficient of drug interaction (CDI). The lowest value CDI = 0.11 ± 0.01 was for the mixture containing half of PBGE-PGGE_1_CLOT_d and PBGE-PGGE_1_NIF_e, for a concentration of 10 μM, and value CDI = 0.14 ± 0.02 for a mixture containing half of PBGE-PGGE_2_CLOT_c and PBGE-PGGE_2_NIF_e, also for a concentration of 10 μM.

Moreover, comparing the IC_50_ values for HMEC-1 for single compounds and combined administration, a decrease of this parameter value can be seen—the cytotoxicity of the combined system administration toward noncancer cells increases. But at the same time, the IC_50_ values for HeLa cancer cells decreased drastically, which means that the selectivity for cancer cells is preserved. Importantly, the increased cytotoxicity against cancer cells allows the use of much lower doses of the carrier containing both drugs compared to drugs encapsulated individually in polymers and even more so compared to free drugs while maintaining the desired therapeutic effect. It is also known that lowering the dose of the drug will reduce the potential side effects of its use. It is worth noting that the most effective mixture used in the coadministration consisted of equal parts of PBGE-PGGE_1_CLOT_d clotrimazole and PBGE-PGGE_1_NIF_e nifuratel and PBGE-PGGE_2_CLOT_c and PBGE-PGGE_2_NIF_e nifuratel, respectively, which means that a 1 mM mixture contained exactly 0.5 mM clotrimazole and 0.5 mM nifuratel. Thus, the IC_50_ values and significant synergy effects obtained do not result from the use of a double amount of drugs.

Comparing the efficacy of both free and encapsulated drugs in star-shaped PBGE-PGGE unimolecular micelles, it can be noted that some systems with clotrimazole increased their efficacy against cancer cells at the expense of reducing their selectivity against noncancer cells, and vice versa in the case of encapsulated nifuratel, which acquired selectivity against cancer cells. At this stage of the research, we cannot clearly explain why nifuratel encapsulated in star-shaped copolyether has a selective effect on cervical cancer cells and is not toxic to noncancerous endothelial cells. It is known from the literature that the effect of nifuratel may depend on the cell type and, more specifically, on the abnormalities that occur in the signaling pathways in these cells.

As mentioned above, Zheng et al. showed that nifuratel can inhibit proliferation and induced apoptosis of gastric cancer cells (cell lines SGC-7901 and BGC-823).^20^ This was due to nifuratel blocking the induction of IL6 activation of the STAT3 signaling pathway. Nifuratel also increased the expression of the proapoptotic protein Bax and decreased the expression of the antiapoptotic protein Bcl-2. Therefore, for all tested compounds, as well as for unloaded star-shaped copolyether and pure drugs, we examined the expression of several selected genes related to cytokines, i.e., *IL1*—a cytokine capable of inducing the secretion of IFN-γ, IL6, or TNF; *TNFa*—tumor necrosis factor and *NFKBIA*, which encodes members of the NF-kappa-B inhibitor.

The results obtained indicate differences in the expression levels of these genes in both cell lines (Tables S1 and S2 with the relative expression of the NFκB pathway and cytokine-related genes in HeLa and HMEC-1 cell lines). Cytokine levels were higher in untreated HMEC-1 cells than in cancerous HeLa cells. Pure nifuratel increased the expression of *NFKBIA* in both cell lines and increased *IL1* expression in HMEC-1, but did not activate *TNFa*. Interestingly, unloaded and nifuratel-loaded star-shaped copolyether did not increase and sometimes decreased the expression level of *NFKBIA*, increased the expression level of *IL1* in cancer cells and decreased it in noncancer cells, and decreased the expression level of *TNFa* in both lines compared to the control. It is well known that blocking the translocation of NF-κB from the cytoplasm to the nucleus enhances the proapoptotic effect of, among others, TNF family cytokines, chemotherapeutics, ionizing radiation, and hormones.^42^ However, the signaling pathways in cancer cells do not function properly, and therefore, NF-κB can also direct many cell types toward apoptosis induced by various factors, as studies by Stark et al. showed that translocation to the nucleus of the NF-κB transcription factor precedes the process of aspirin-induced apoptosis.^43^ Therefore, we are inclined to the hypothesis, which requires further research, that the mechanism of action of encapsulated nifuratel is related to the inhibition of NF-κB and cytokine pathways, while its selectivity results from differences in the signaling pathways of cancer and noncancerous cells and its combined action with star-shaped copolyethers.

Since selected genes are responsible not only for programmed cell death but also for the proinflammatory response in cells, the lack of their activation by drugs encapsulated in star-shaped PBGE-PGGE unimolecular micelles compared to free drugs may indicate their immunological inertness, which is important information proving the biocompatibility of the tested compounds. Summarizing the entire panel of performed methods, in view of future planned *in vivo* studies and intratumoral administration of the studied systems, we also decided to perform hemolysis tests on blood cells from healthy donors since we could not exclude the spread of the encapsulated drugs from the site of administration through blood vessels.

**Figure 9:**
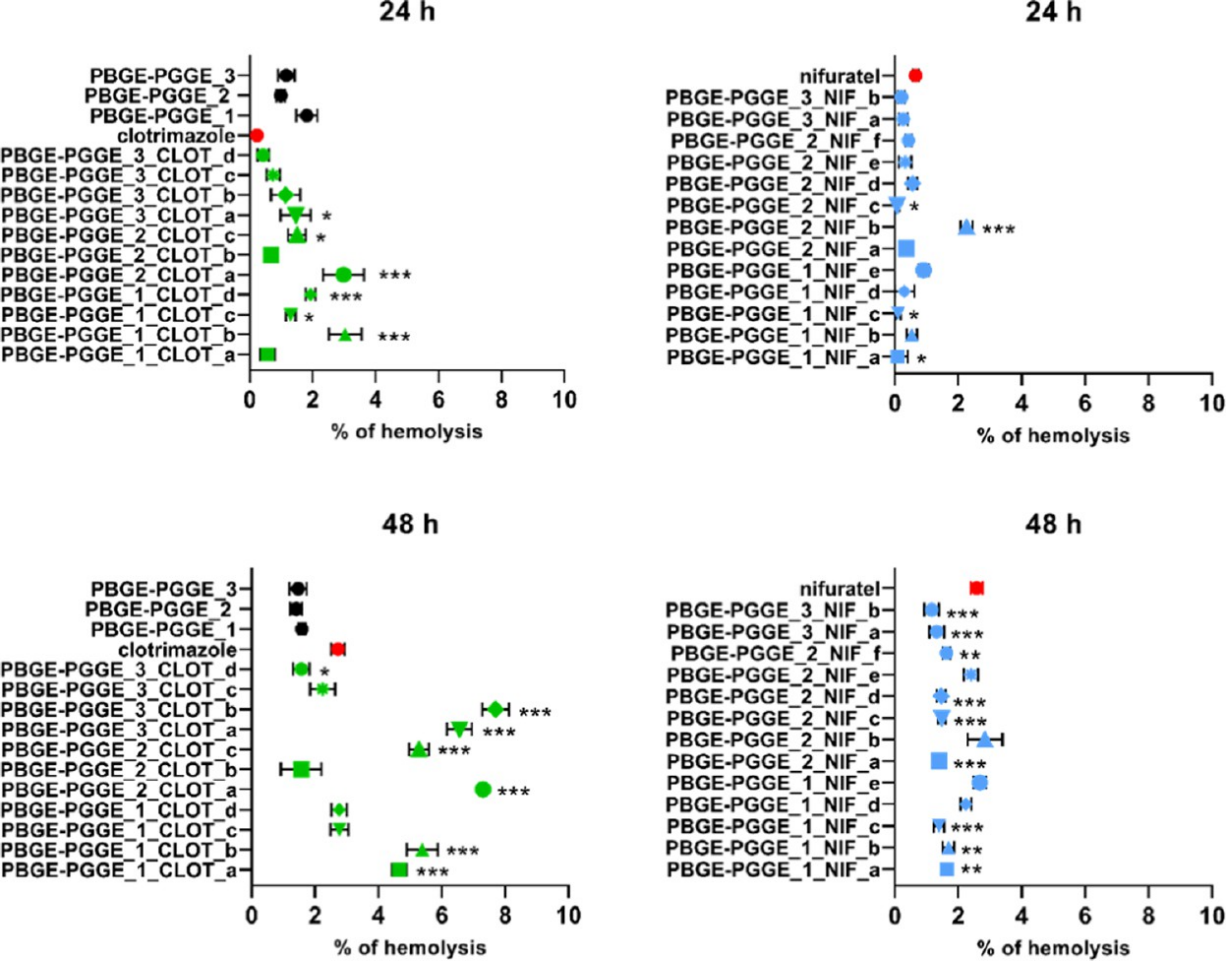
Hemolytic activity of clotrimazole and PBGE-PGGE star-shaped copolyethers with/without the drug in the concentration range of 0.1–100 μM after 24 and 48 h of incubation (left panel) and the hemolytic activity of nifuratel and PBGE-PGGE star-shaped copolyethers with the drug in the concentration range of 0.1–100 μM after 24 and 48 h of incubation (right panel). The results are presented as the mean ± SD (*n* = 4). The results were considered significant at *p ≤ 0.01, **p ≤ 0.001, and ***p ≤ 0.0001 with regard to clotrimazole or nifuratel, respectively.

After isolation of red blood cells from the blood of a healthy donor, samples with a hematocrit of 2% were incubated with the tested drugs and PBGE-PGGE star-shaped copolyethers with/without drugs in the concentration range of 0.1–100 μM for 24 and 48 h, respectively. All partial results, together with the statistical analysis, are presented in two tables in the Supporting Information (Tables S3 and S4). The most interesting results indicating the absence or low hemotoxicity of the tested compounds are presented in Figure 9. Even after 48 h of incubation, unloaded PBGE-PGGE star-shaped copolyethers caused less than 2% hemolysis, lower than that observed for free drugs. Administration of encapsulated clotrimazole at the highest concentration of 100 μM increased hemolysis by up to 5% for the most anticancer-effective systems. Conversely, administration of encapsulated nifuratel at the same concentration did not increase the level of hemolysis, which in most cases was even statistically significantly lower than for the drug itself. The results obtained allow the safe use of the tested compounds in *in vivo* tests.

Since both described above systems can also be obtained in the form of hydrogels, for our research and this article, we decided to create an experiment simulating the application of the gel to the surface of the cervix attacked by Hela cancer cells. The concentration of drugs in the hydrogel platforms is given in Table 7.

**Table 7:** Concentration of Nifuratel and Clotrimazole Loaded in PBGE-PGGE-Based Hydrogels

hydrogel	nifuratel concentration, mmol_drug_/g_hydrogel_	clotrimazole concentration, mg_drug_/g_hydrogel_
PBGE-PGGE_1/P(AM-2-AAPBA)_neat		
PBGE-PGGE_1_NIF_e/P(AM-2-AAPBA)	0.0264	
PBGE-PGGE_1_CLOT_d/P(AM-2-AAPBA)		0.0144
PBGE-PGGE_1_NIF_e/PBGE-PGGE_1_CLOT_d/P(AM-2-AAPBA)	0.0264	0.0144
PBGE-PGGE_2/P(AM-2-AAPBA)_neat		
PBGE-PGGE_2_NIF_e/P(AM-2-AAPBA)	0.0154	
PBGE-PGGE_2_CLOT_c/P(AM-2-AAPBA)		0.0288
PBGE-PGGE_2_NIF_e/PBGE-PGGE_2_CLOT_c/P(AM-2-AAPBA)	0.0154	0.0288

**Figure 10:**
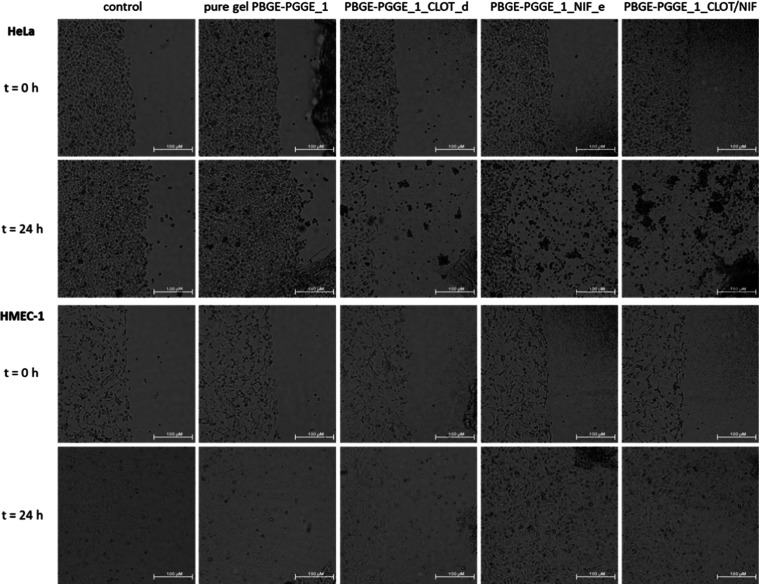
Influence of the neat hydrogel (PBGE-PGGE_1), the hydrogel with clotrimazole (PBGE-PGGE_1_CLOT_d), the hydrogel with nifuratel (PBGE-PGGE_1_NIF_e), and the hydrogel containing equal parts of both drugs (PBGE-PGGE_1_CLOT/NIF) on HeLa (upper panel) and HMEC-1 (bottom panel) cell viability immediately after gel administration and after 24 h of incubation. Microscope magnification 4×, bar 100 μm.

**Figure 11:**
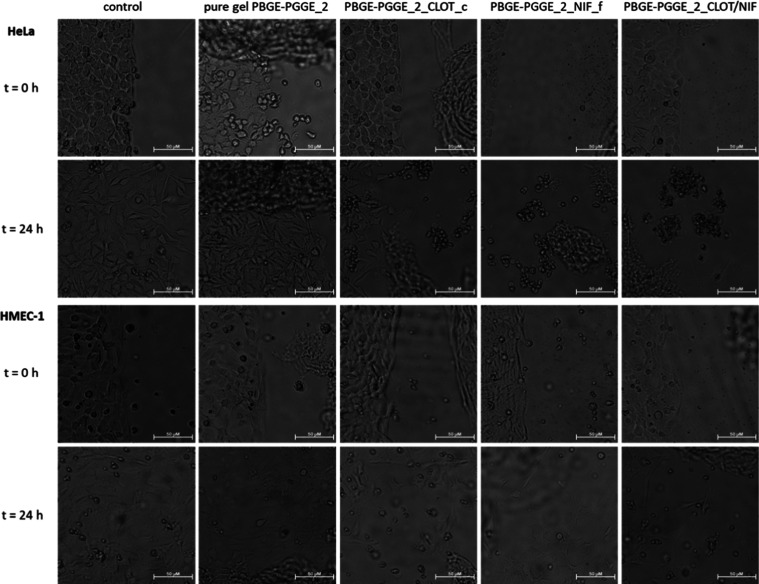
Influence of the neat hydrogel (PBGE-PGGE_2), the hydrogel with clotrimazole (PBGE-PGGE_2_CLOT_c), the hydrogel with nifuratel (PBGE-PGGE_2_NIF_e), and the hydrogel containing equal parts of both drugs (PBGE-PGGE_2_CLOT/NIF) on HeLa (upper panel) and HMEC-1 (bottom panel) cell viability immediately after gel administration and after 24 h of incubation. Microscope magnification 4×, bar 50 μm.

Briefly, the cells were plated on 12-well transparent plates with the cell culture insert ibidi, but in our experiment, we allowed the cells to grow freely on the well surface outside the insert while an appropriate amount of gel was squeezed into both dry chambers of the cell culture insert to allow it to adhere to the clean surface of the plate. After removing the inset, the cells and the gel portions were separated by a distance of about 100 nm. The cells, not being directly covered with the gel, were not cut off from the supply of the culture medium and were able to divide freely. Observation of drug-loaded micelles releasing from the gel and cellular response was observed immediately after removal of the cell culture insert and after 24 h of incubation. The influence of hydrogel systems, i.e., neat, single, and both drug-loaded systems, on the HeLa and HMEC-1 cell lines is visualized in Figures 10 and 11. To facilitate the evaluation of the microscopic images (Figures 10 and 11), we decided to use the ImageJ program1 to assess the area with the cells and determine its increase or decrease caused by the action of the tested gels (Tables S5 and S6).

The hydrogel constructed of the neat PBGE-PGGE_1 copolymer did not affect cell division and migration (Figure 10). The hydrogel constructed of clotrimazole-loaded PBGE-PGGE_1, i.e., PBGE-PGGE_1_CLOT_d, after 24 h of incubation, caused the death of a large part of the HeLa cancer cell population and slowed down the division of noncancerous HMEC-1 cells. This is not consistent with the results obtained by the MTT method—the clotrimazole encapsulated in the hydrogel was more effective compared to its aqueous solution (in PBS). The nifuratel-loaded hydrogel PBGE-PGGE_1, i.e., PBGE-PGGE_1_NIF_e, according to the results obtained by the MTT method, after 24 h of incubation, caused the death of a greater part of HeLa cancer cell population but did not slow down the division of noncancerous HMEC-1 cells. As expected, the hydrogel consisting of equal parts of clotrimazole PBGE-PGGE_1_CLOT_d and nifuratel PBGE-PGGE_1_NIF_e caused the death of all HeLa cancer cells but did not slow down the division of noncancerous HMEC-1 cells, which is consistent with the results obtained by the MTT method—the drugs incorporated into the hydrogels were more effective compared to their aqueous solution (in PBS). The drug-free PBGE-PGGE_2 hydrogel also did not affect the rate of cell division and migration, but from its structures, the drugs were released more slowly. Similarly, we observed a discrepancy between the MTT results for aqueous solutions of drugs encapsulated in the PBGE-PGGE_2 polymer and the images of their counterparts in the gel formulation. Hydrogels loaded with single drugs (especially with nifuratel) were more cytotoxic to tumor cells. The MTT results of the PBGE-PGGE_2_CLOT/NIF coadministration are consistent with the images obtained only for Hela cells—where the death of all cancer cells can be observed. Images of noncancerous HMEC-1 cells show that some cells continue to proliferate, which is no longer consistent with the MTT results. In conclusion, in both groups of PBGE-PGGE_1 and PBGE-PGGE_2, hydrogel formulations are significantly more effective and show greater selectivity compared to their respective aqueous solutions prepared in PBS buffer.

### 3.7 Bioadhesion of PBGE-PGGE-Based Hydrogels

**Figure 12:**
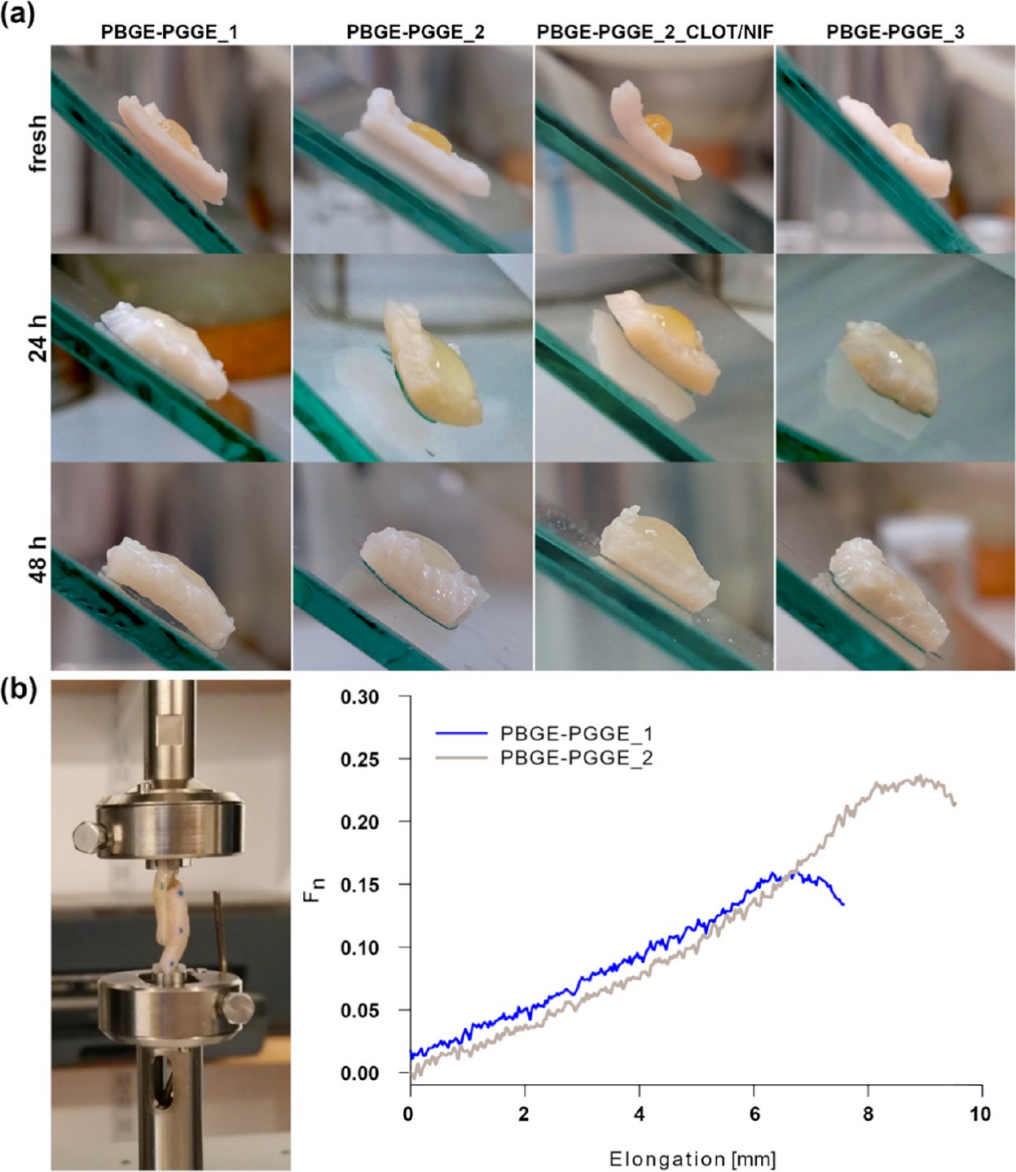
(a) Behavior of free- and drug-loaded PBGE-PGGE_2 hydrogels on the porcine skin incubated in a simulated vaginal fluid for 24 and 48 h at 37 °C. (b) Lap shear tests performed for hydrogels constructed of reversibly cross-linked PBGE-PGGE differing in the ratio of DP*_n_* (shell)/DP*_n_* (core).

With regard to the intended use of the designed hydrogels to deliver drugs to the afflicted tissue areas, we investigated the bioadhesion ability of star-shaped copolyether-based hydrogels to the tissue in the vagina environment. The proper adhesion of the drug carrier to the tissue in wet conditions is important to ensure prolonged contact with bioactive substances. Bioadhesion of hydrogels at the aqueous conditions cannot be too high to avoid uncontrolled sticking of the tissue. The adhesion strength of synthesized networks was performed using the lap shear test for PBGE-PGGE_1 and PBGE-PGGE_2 hydrogels to SVF-saturated porcine skin. The bioadhesive strength ranged from 1.5 to 2.1 kPa for PBGE-PGGE_1 and PBGE-PGGE_2 hydrogels, respectively (Figure 12b). The behavior of PBGE-PGGE hydrogels deposited on porcine skin at vaginal conditions (SVF) was visualized based on neat and drug-loaded hydrogels constructed of PBGE-PGGE_2 (Figure 12a). The structure of the hydrogels constructed of PBGE-PGGE_1 and PBGE-PGGE_2 copolymers was stable for 48 h, with visible swelling in the case of the hydrogel with PBGE-PGGE_2. There was no difference in structure stability behavior in the presence of drugs. In the case of the hydrogel constructed with PBGE-PGGE_3, swelling of the network was visible after 24 h, with its significant decay visible after 48, indicating rapid degradation of the polymer network, and thus further study of this hydrogel was not continued.

### 3.8 *In Vitro* Study of Artificial Skin Permeability with Hydrophobic Drugs Loaded in PBGE-PGGE Unimolecular Micelles

**Figure 13:**
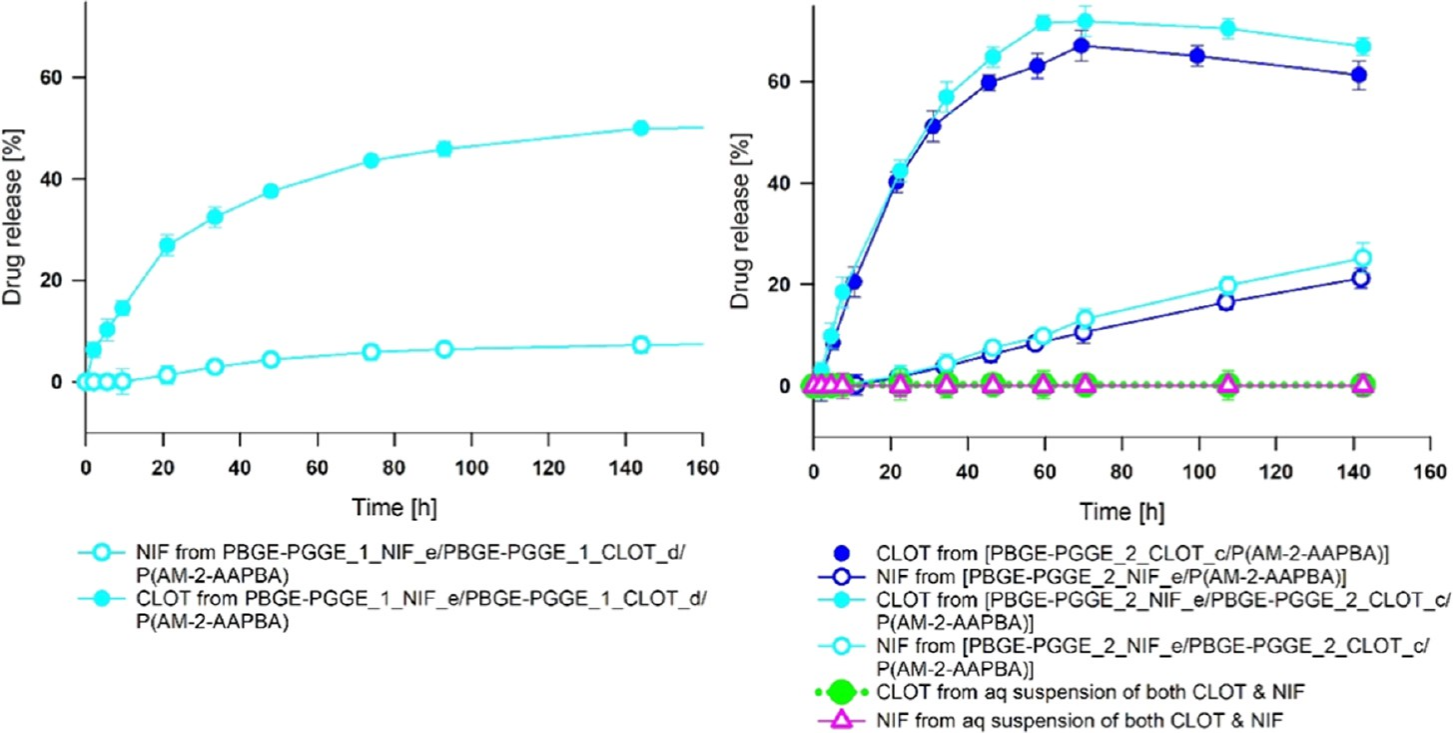
*In vitro* study of permeability via the Strat-M membrane of drugs loaded in hydrogels constructed of PBGE-PGGE_1 (on the left) and PBGE-PGGE_2 (on the right) compared with free drugs suspended in water.

Due to promising biological results obtained for hydrogel-based drug delivery systems, we performed *in vitro* permeability experiments of drugs across a Strat-M membrane, i.e., a synthetic nonanimal model for transdermal diffusion, which is used to predict the diffusion of drug molecules through the skin, mounted in the Franz diffusion cell, using drug-loaded hydrogel carriers and an aqueous suspension of pure drugs. The study revealed that the permeation of drugs through the membrane is significantly increased in the case of drugs incorporated into the hydrogel matrix (Figure 13). Undoubtedly, the hydrogel formulation facilitates the transfer of hydrophobic drugs across the membrane. However, nifuratel displayed lower permeability through the membrane in comparison to clotrimazole. Drugs suspended in the aqueous medium did not effectively penetrate the membrane. The permeability constant, *K*_p_, is given as follows
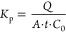
3where *Q* is the amount of the drug transported through the membrane in time *t*, *A* is the area exposed membrane, and *C*_0_—donor concentration, determined for both drugs enclosed in the hydrogel matrix, was approximately 90 times higher than it was observed for drugs in the aqueous suspension of drugs (Table 8). The flux, *J*
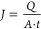
4of clotrimazole and nifuratel enclosed in the PBGE-PGGE_2-based hydrogel was 6.50 × 10^–5^ and 5.50 × 10^–6^ mg/cm^2^/min, respectively, whereas the flux of drugs from the aqueous suspension was only 3.55 × 10^–6^ and 2.61 × 10^–8^ mg/cm^2^/min for clotrimazole and nifuratel, respectively. In addition, based on the comparison of the permeability constants of drugs incorporated in both a single- and combination therapy, no effect of the accompanying drug on the flux of the second drug and its permeability constant through the membrane was observed (Table 8).

**Table 8:** Comparison of the Permeability Constant (*K*_p_) of Clotrimazole and Nifuratel Loaded in the Hydrogel Carriers and Suspended in an Aqueous Medium through the STRAT-M Membrane

matrix	*K*_p_ clotrimazole, mg/cm^2^/min	*K*_p_ nifuratel, mg/cm^2^/min
PBGE-PGGE_1_NIF_e/PBGE-PGGE_1_CLOT_d/P(AM-2-AAPBA)	5.75 × 10^–5^	2.72 × 10^–6^
PBGE-PGGE_2_NIF_e/PBGE-PGGE_2_CLOT_c/P(AM-2-AAPBA)	6.74 × 10^–5^	2.90 × 10^–6^
PBGE-PGGE_2_CLOT_c/P(AM-2-AAPBA)	6.03 × 10^–5^	
PBGE-PGGE_2_NIF_e/P(AM-2-AAPBA)		2.42 × 10^–6^
Pure CLOT/NIF	6.73 × 10^–7^	2.75 × 10^–8^

Both bioadhesion and permeability studies undoubtedly input that the hydrogel platform based on PBGE-PGGE unimolecular micelles dynamically cross-linked via boronic ester cross-links can ensure sustained drug delivery to the afflicted area.

## 4 Conclusions

In summary, for the first time, hydrogel drug carriers were constructed to surmount the challenges of the lack of treatment selectivity that current anticervical cancer therapies suffer from.

Hydrogels were constructed of drug-loaded unimolecular micelles based on star-shaped block amphiphilic copolyethers with the distinctive hydrophobic core of poly(benzyl glycidyl ether) and the hydrophilic shell composed of poly(glyceryl glycerol ether). From one side, these unimolecular micelles ensure the maintenance of the selective anticancer activity of clotrimazole. On another side, nifuratel has acquired selective anticancer properties due to its encapsulation inside unimolecular micelles. Unexpectedly, by combining unimolecular micelles loaded with two drugs, a significant synergistic anticervical cancer activity was obtained. In addition, the cross-linking of such drug-loaded unimolecular micelles via dynamic covalent bonds based on a diol-boronic acid reaction resulted in the formation of hydrogels that also displayed significant synergistic anticancer behavior for combination therapy. The usage of dynamic boronic ester bonds to cross-link unimolecular micelles provided the injectable and self-healable hydrogel drug platforms, which can be conveniently administered via an intravaginal route, and ensured sustained drug delivery to the afflicted tissue area, facilitating its permeability for encapsulated clotrimazole and nifuratel, in contrast to pure drugs. Here, we show that the combination of star-shaped polyether amphiphiles and boronic ester cross-linking chemistry provides a new strategy for obtaining hydrogel platforms suitable for efficient hydrophobic drug delivery.

It is the first report in which we focus on the usage of unimolecular micelles to enhance the bioavailability of water-insoluble clotrimazole and nifuratel by increasing their solubilization in the aqueous environment, demonstrating the enhancement of their anticancer activity. This approach allows to reduce the dose of the drugs while maintaining the therapeutic efficacy.

## Supporting Information Available

PBGE-PGGE_1_hydrogel (MP4)
PBGE-PGGE_2_hydrogel (MP4)
PBGE-PGGE_3_hydrogel (MP4)
^1^H NMR spectra for all copolymers, additional rheological results, and cytotoxicity and hemotoxicity data (PDF)
