# Supplementary material for: Selective Anticervical Cancer Injectable and Self-Healable Hydrogel Platforms Constructed of Drug-Loaded Cross-Linkable Unimolecular Micelles in a Single and Combination Therapy
Source: ACS Appl Mater Interfaces. 2024 Mar 15;16(12):14605–25. doi: 10.1021/acsami.4c01524 (PMC10982937; doi:10.1021/acsami.4c01524)
Supplement: Supplementary file 4 — am4c01524_si_004.pdf [file am4c01524_si_004.pdf]

Selective anti-cervical cancer injectable and self-healable hydrogel platforms constructed of drug-loaded cross-linkable unimolecular micelles in a single and combination therapy

Monika Gosecka[a], Mateusz Gosecki,[a] Piotr Ziemczonek,[a] Malgorzata Urbaniak,[a] Ewelina Wielgus,[a] Monika Marcinkowska, [b] Anna Janaszewska*[b], Barbara Klajnert-Maculewicz [b]*

a. Centre of Molecular and Macromolecular Studies, Polish Academy of Sciences Sienkiewicza 112, 90-363 Lodz, Poland

* Correspondence: mdybko@cbmm.lodz.pl

b. Department of General Biophysics, Faculty of Biology and Environmental Protection, University of Lodz, 141/143 Pomorska Street, 90-236 Lodz, Poland

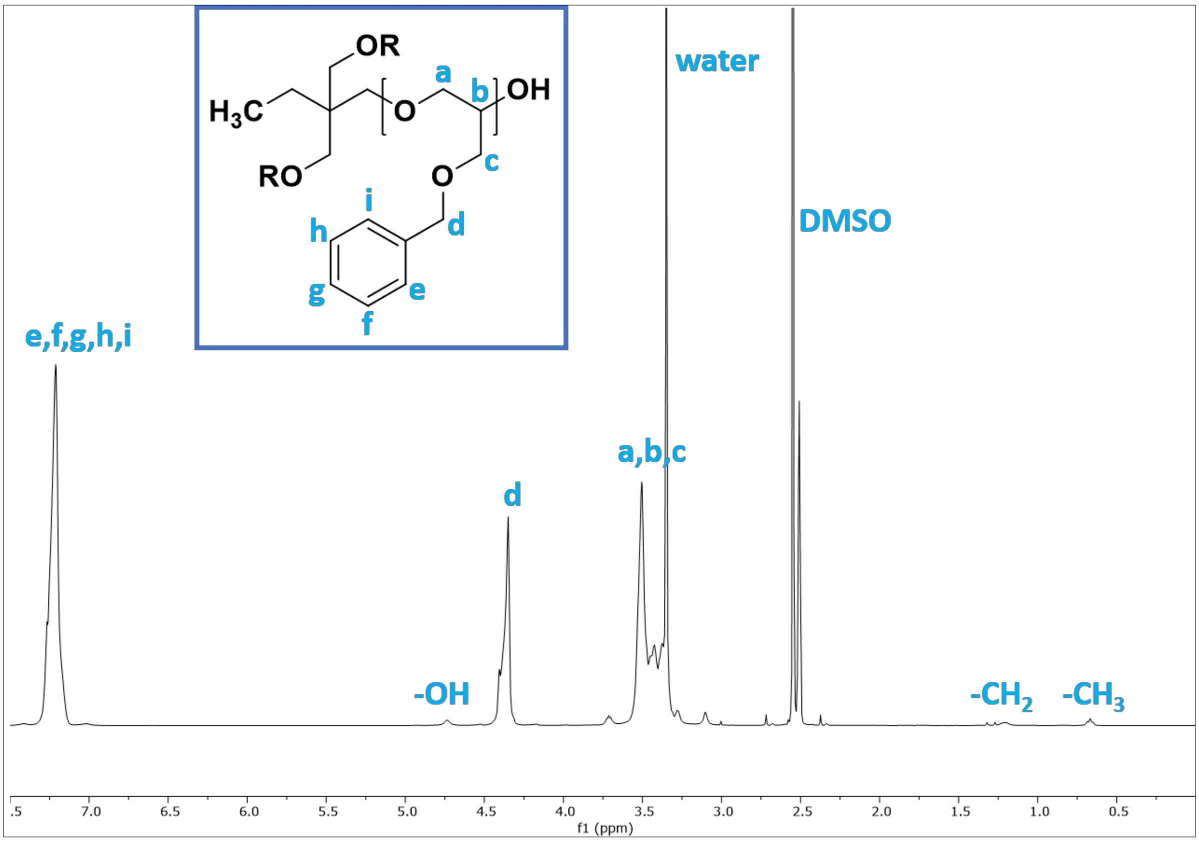

Figure S1. ^{1}H NMR spectrum of PBGE_15 recorded in DMSO-d_6.

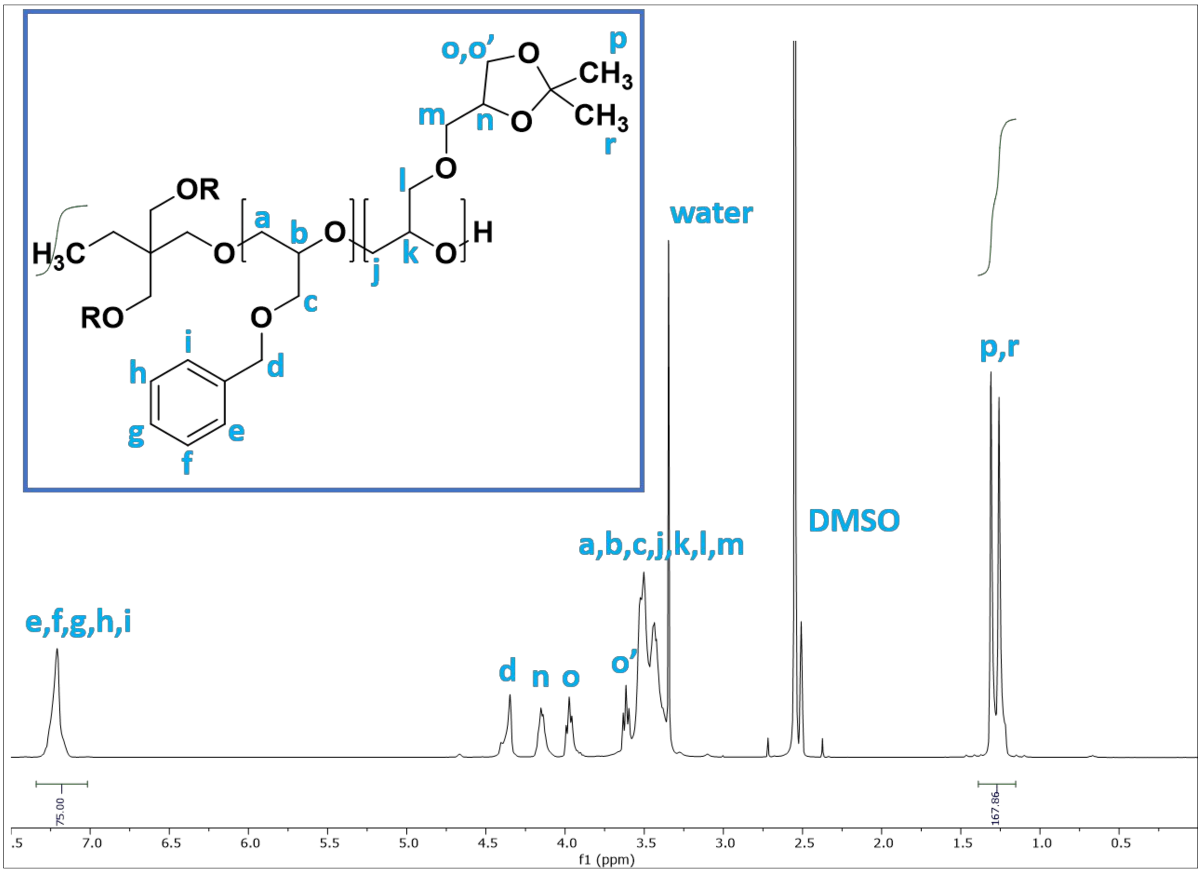

Figure S2. ^{1}H NMR spectrum of PBGE-PIGE_15/30 recorded in DMSO-d_6.

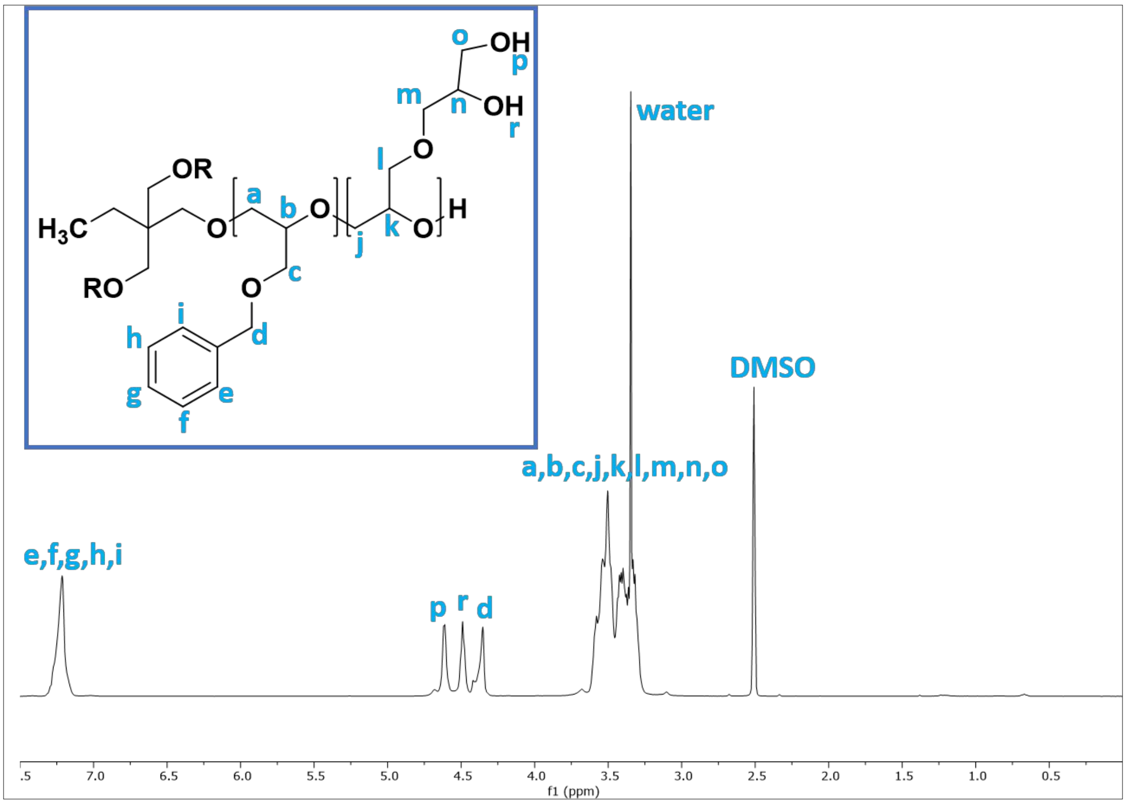

Figure S3. ^{1}H NMR spectrum of PBGE-PGGE_15/30 recorded in DMSO-d_6.

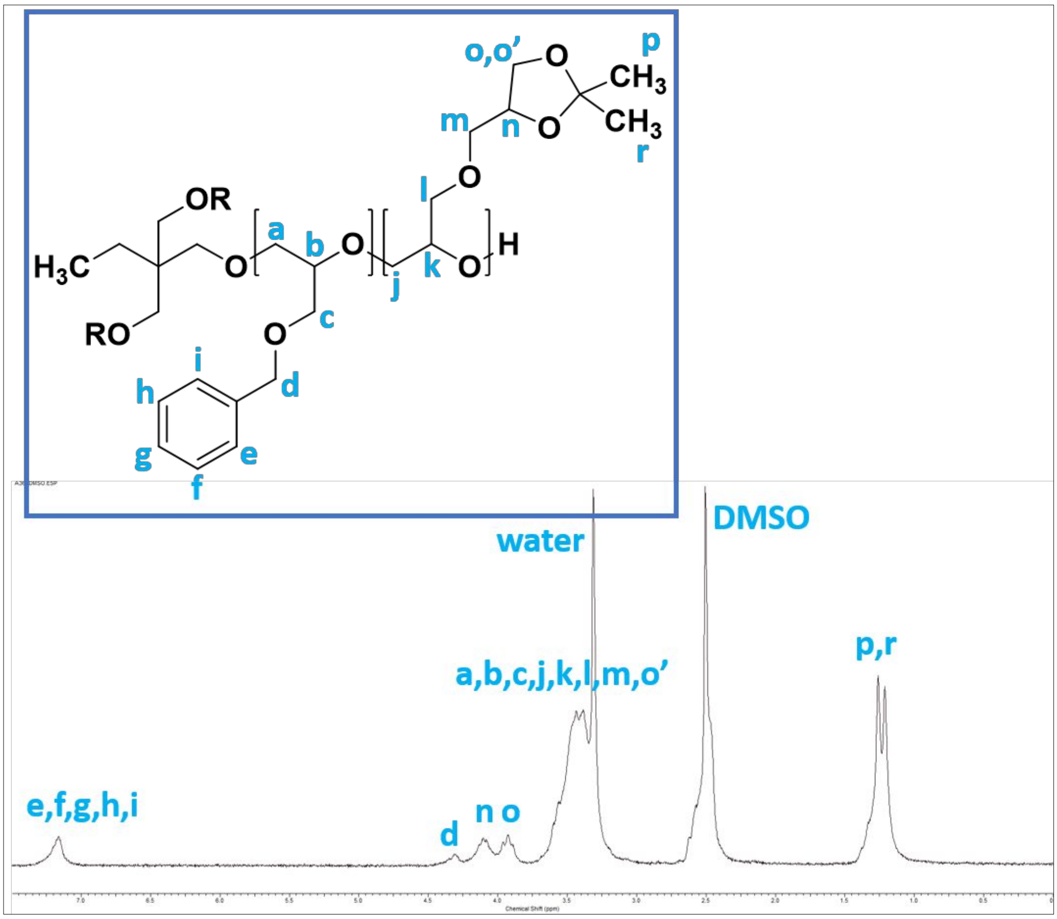

Figure S4. ^{1}H NMR spectrum of PBGE-PIGE_15/86 recorded in DMSO-d_6.

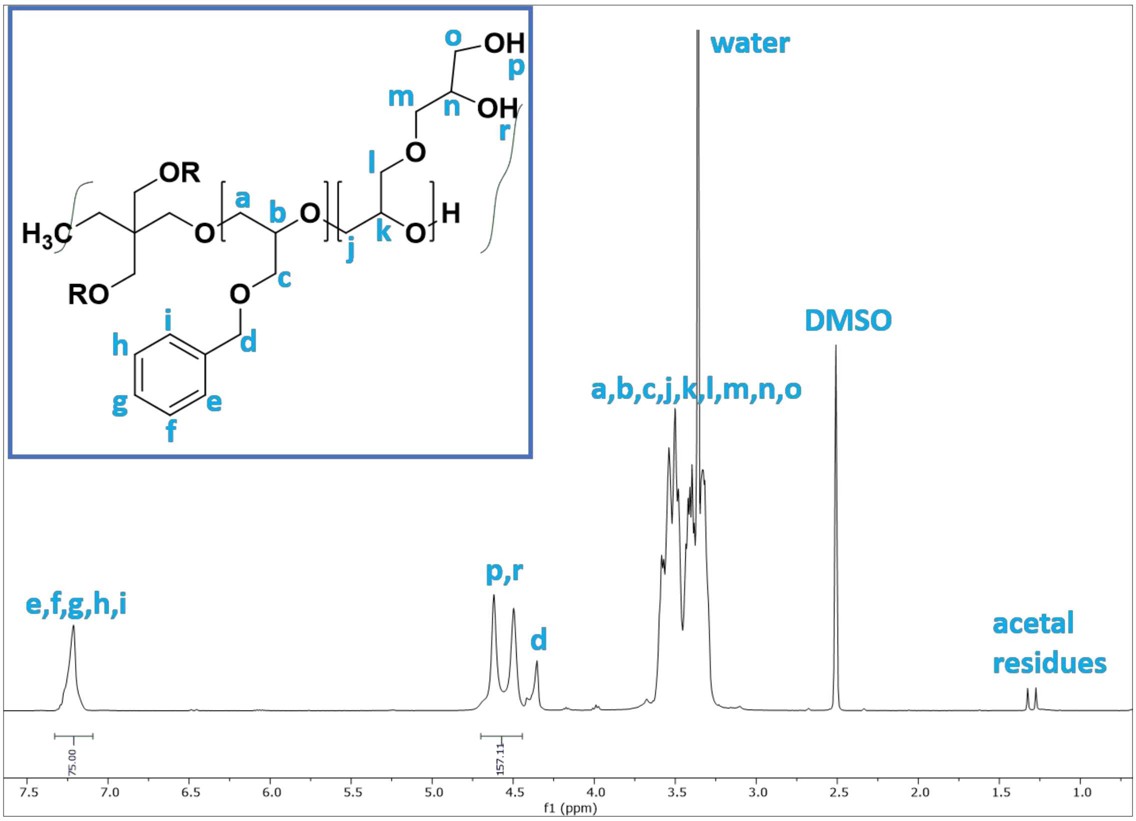

Figure S5. ^{1}H NMR spectrum of PBGE-PGGE_15/86 recorded in DMSO-d_6.

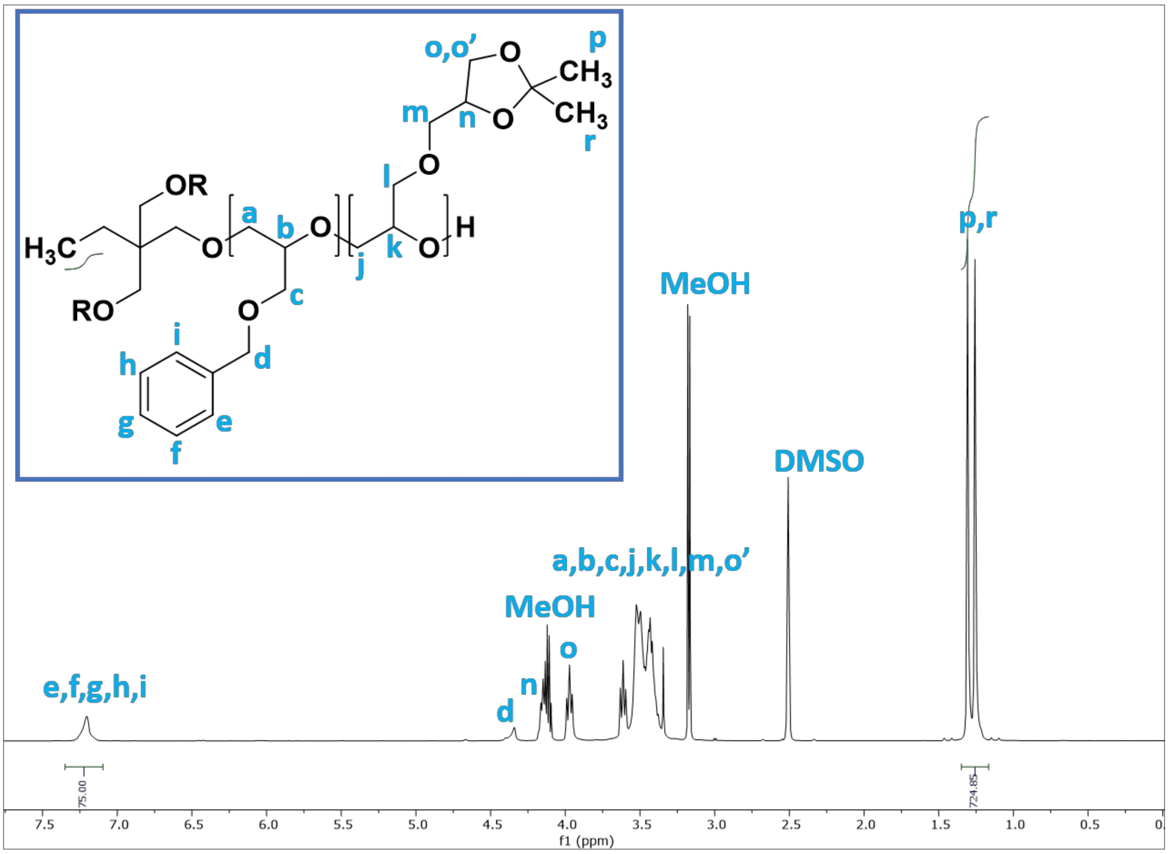

Figure S6. ^{1}H NMR spectrum of PBGE-PIGE_15/120 recorded in DMSO-d_6.

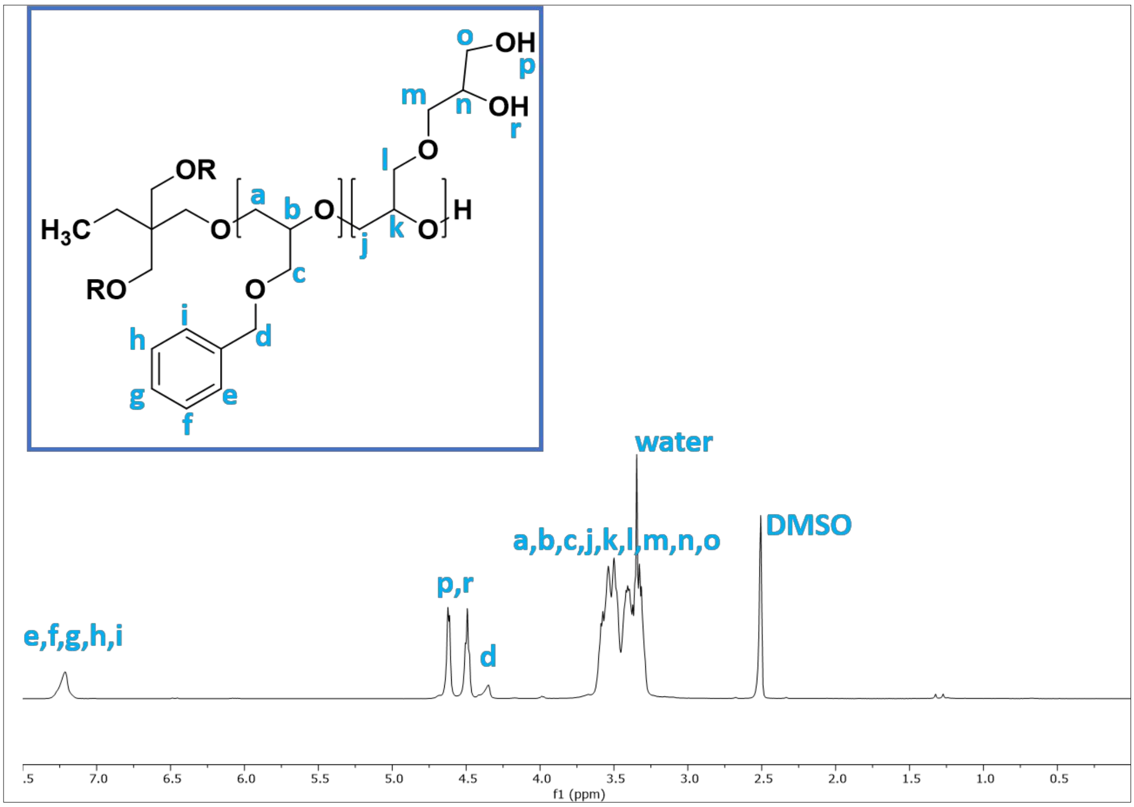

Figure S7. ^{1}H NMR spectrum of PBGE-PGGE_15/120 recorded in DMSO-d_6.

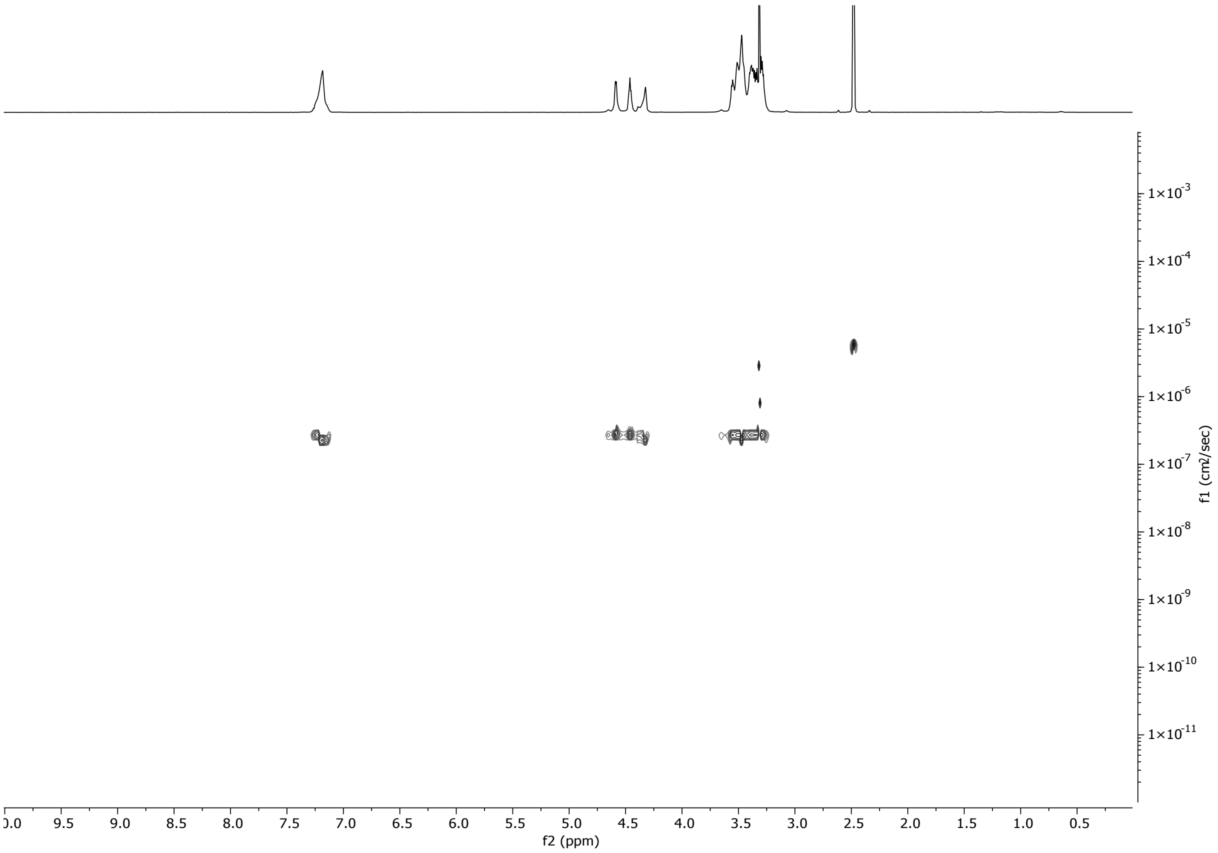

Figure S8. ^{1}H DOSY NMR spectrum of PBGE-PGGE_15/30 recorded in DMSO-d_6.

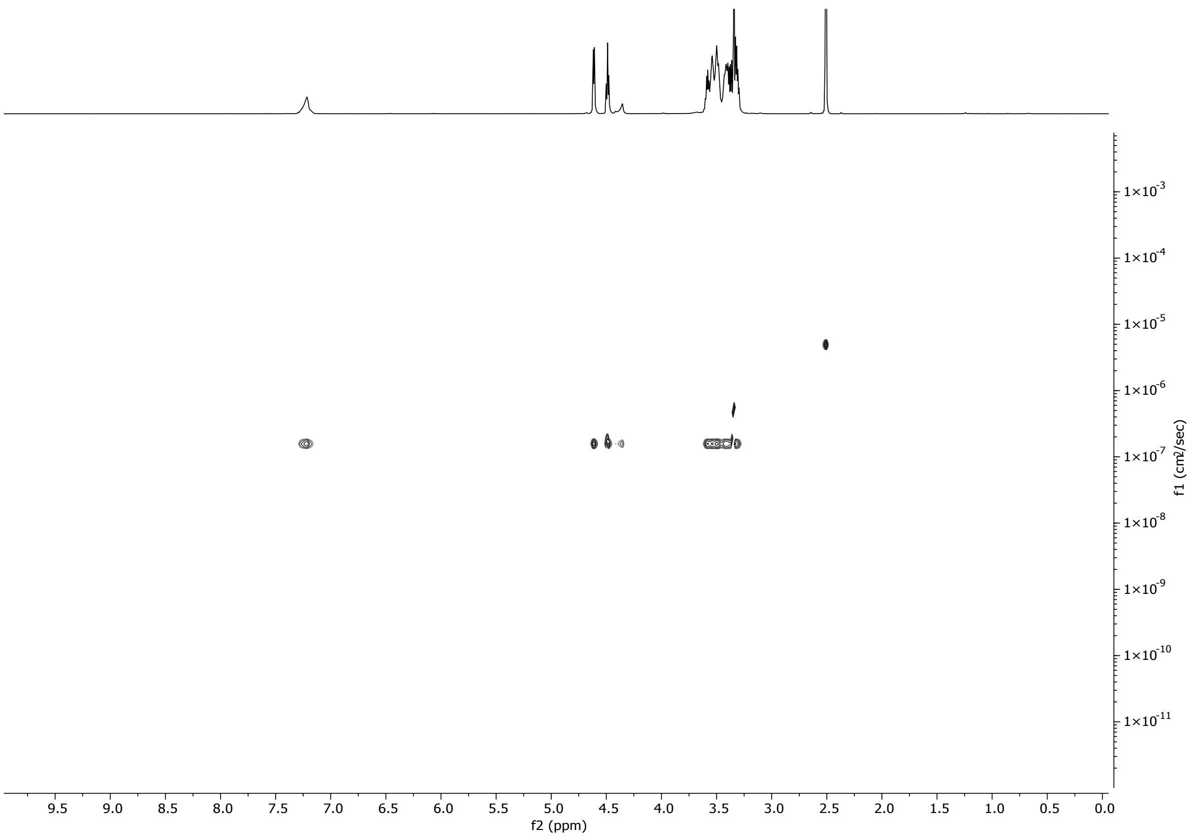

Figure S9. ^{1}H DOSY NMR spectrum of PBGE-PGGE_15/86 recorded in DMSO-d_6.

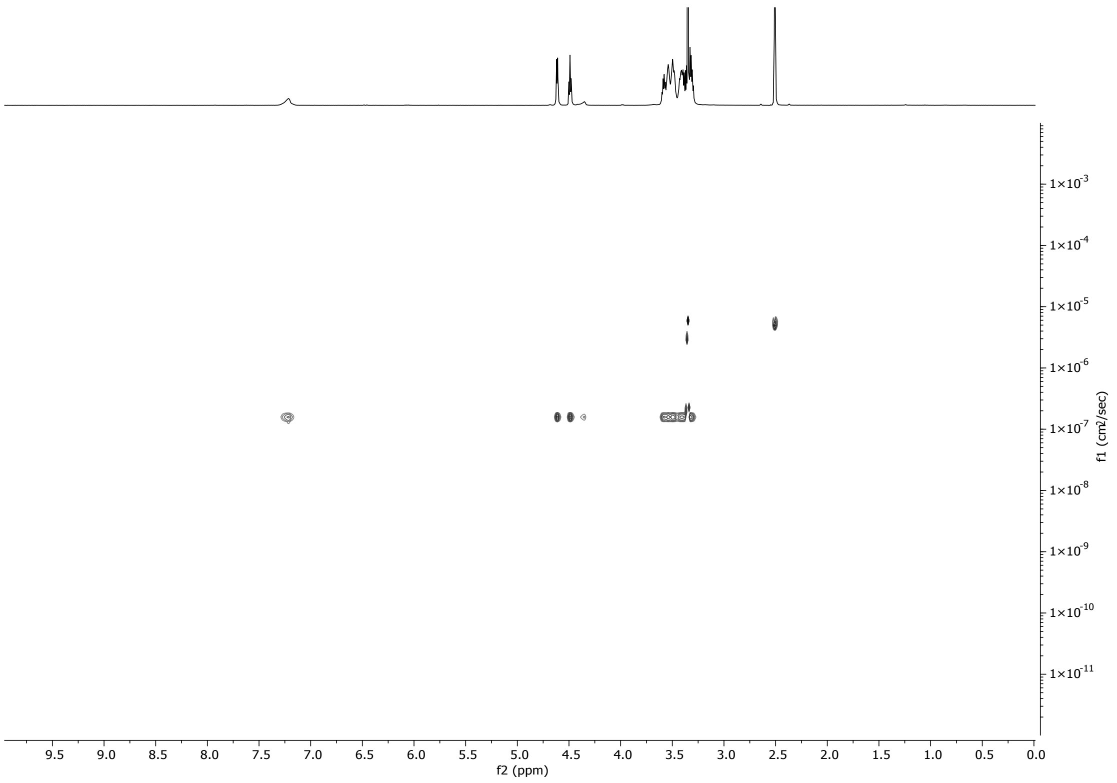

Figure S10. ^{1}H DOSY NMR spectrum of PBGE-PGGE_15/120 recorded in DMSO-d_6.

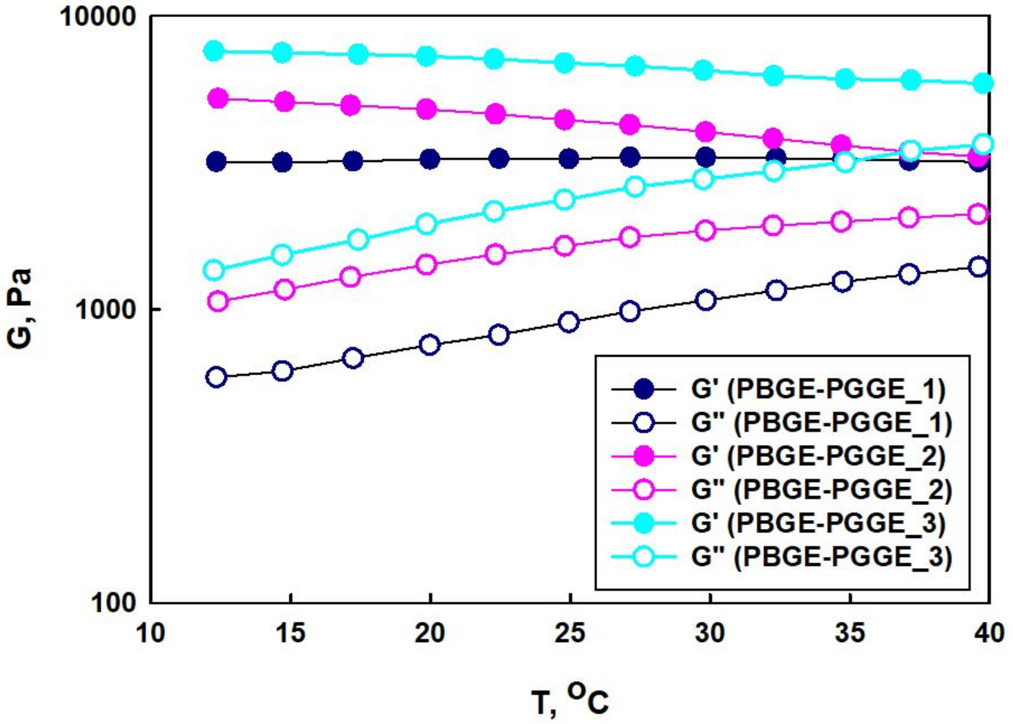

Figure S11. Temperature dependence of moduli recorded for hydrogels (17 wt%) constructed of PBGE-PGGE copolymers differing the length of the hydrophilic shell.

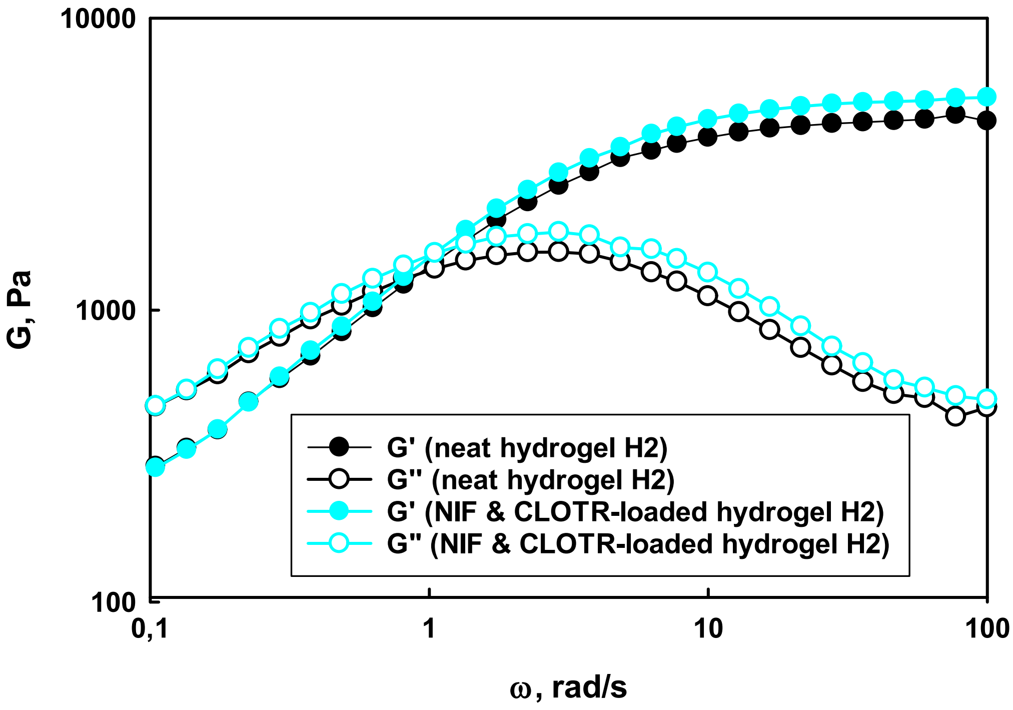

Figure S12. Frequency sweep experiments performed for drug-free, and both nifuratel and clotrimazole-loaded hydrogel based on PBGE-PGGE_2 at 25 °C. ($C_{nifuratel}$ = 0.0154 mmol per g of hydrogel; $C_{clotrimazol}$ = 0.029 mmol per g of hydrogel).

The expression level of NF-κB marker gene *NFKBIA* and cytokine genes *IL1*, *TNFa* was determined by quantitative real-time RT-PCR. Due to the lack of literature data on the effect of nifuratel or clotrimazole on the activity of selected genes in the cancerous HeLa line and the non-cancerous HMEC-1 line, as a first step, we performed an experiment to evaluate the changes in the expression level of selected genes as a function of time, after administration of 10 μM pure drugs. The results are shown in **Table S1**. Based on the first experiment, a time of 120 min (time after which activation of all selected genes was observed) was chosen for further determinations with drugs encapsulated in star-shaped copolyethers based on PBGE-PGGE - the 10 μM concentration was kept as optimal (no dead cells were observed). The results are shown in **Table S1**.

Table S1. Relative expression of NF-κB pathway and cytokine-related genes in HeLa (left panel) and HMEC-1 (right panel) cell lines. The results are presented as the mean ± SD, n = 3. The results were considered significant at *p ≤ 0.0001 compared to untreated control cells.

**HeLa**	*NFKBIA*	*IL1*	*TNFa*	**HMEC-1**	*NFKBIA*	*IL1*	TNFa
**untreated cells**	**0.16 ± 0.13**	**0.01 ± 0.49**	**5.01 ± 0.32**		**12.99 ± 0.12**	**6.42 ± 0.23**	**9.31 ± 0.32**
clotrimazole 15 min	4.99 ± 0.09*	0.04 ± 0.24	0.04 ± 0.30*		13.64 ± 0.28	74.86 ± 0.20*	0.23 ± 2.44*
clotrimazole 30 min	1.60 ± 0.12*	0.02 ± 0.55	0.03 ± 0.46*		15.94 ± 1.28*	114.78 ± 0.11*	0.30 ± 0.23*
clotrimazole 60 min	1.82 ± 0.10*	0.03 ± 0.39	0.02 ± 0.05*		16.31 ± 0.24*	70.57 ± 0.05*	0.13 ± 0.53*
clotrimazole 90 min	3.50 ± 0.16*	0.08 ± 0.48	0.05 ± 0.42*		19.90 ± 0.11*	53.95 ± 0.13*	0.12 ± 0.23*
clotrimazole 120 min	5.99 ± 0.07*	0.07 ± 0.55	0.01 ± 0.03*		27.44 ± 0.14*	32.23 ± 0.03*	0.23 ± 0.11*

clotrimazole 150 min	4.17 ± 0.14*	0.03 ± 0.58	0.03 ± 0.35*	32.74 ± 0.10*	20.95 ± 0.11*	0.10 ± 0.05*
nifuratel 15 min	4.34 ± 0.13*	0.03 ± 0.01	0.02 ± 0.13*	8.18 ± 0.37*	95.34 ± 0.47*	0.40 ± 0.24*
nifuratel 30 min	3.82 ± 0.09*	0.03 ± 0.02	0.02 ± 0.17*	8.18 ± 0.07*	21.10 ± 0.41*	0.04 ± 0.20*
nifuratel 60 min	5.42 ± 0.58*	0.05 ± 0.04	0.03 ± 0.79*	8.07 ± 0.10*	34.22 ± 0.10*	0.19 ± 0.11*
nifuratel 90 min	4.79 ± 0.18*	0.05 ± 0.03	0.04 ± 0.76*	25.18 ± 0.20*	30.32 ± 0.01*	0.52 ± 0.05*
nifuratel 120 min	6.95 ± 0.07*	0.08 ± 0.13	0.04 ± 0.61*	22.72 ± 0.10*	30.59 ± 0.14*	0.85 ± 0.13*
nifuratel 150 min	7.16 ± 0.10*	0.12 ± 0.16	0.08 ± 0.66*	19.33 ± 0.47*	21.55 ± 0.03*	0.52 ± 0.11*

Table S2. Relative expression of NF-κB pathway and cytokine-related genes in HeLa (left panel) and HMEC-1 (right panel) cell lines. The results are presented as the mean ± SD, n = 3. The results were considered significant at *p ≤ 0.01, **p ≤ 0.001, ***p ≤ 0.0001 compared to untreated control cells, □p ≤ 0.01, □□p ≤ 0.001, □□□p ≤ 0.0001 with regard to clotrimazole and □p ≤ 0.01, □□p ≤ 0.001, □□□p ≤ 0.0001 with regard to nifuratel.

	**HeLa**			**HMEC-1**		
	NFKBIA	*IL1*	*TNFa*	*NFKBIA*	*IL1*	*TNFa*
**untreated cells**	**0.16 ± 0.13**	**0.01 ± 0.49**	**5.01 ± 0.32**	**12.99 ± 0.12**	**6.42 ± 0.23**	**9.31 ± 0.32**
PBGE-PGGE_1	7.69 ± 0.04***	0.12 ± 0.93	1.06 ± 3.30*	31.06 ± 0.23***	9.22 ± 0.12***	0.01 ± 0.23***
PBGE-PGGE_2	5.34 ± 0.86***	0.12 ± 0.05	0.00 ± 0.48**	40.54 ± 0.38***	20.14 ± 0.19***	0.25 ± 0.11***
PBGE-PGGE_3	13.06 ± 0.30***	0.19 ± 1.09	0.62 ± 2.66*	24.25 ± 0.19***	12.36 ± 0.21***	0.09 ± 0.22***

**clotrimazole**	**5.99 ± 0.07*****	**0.07 ± 0.55**	**0.01 ± 0.03****	**27.44 ± 0.14*****	**32.23 ± 0.03*****	**0.23 ± 0.11*****
PBGE-PGGE_1_CLOT_a	5.38 ± 0.20***	0.05 ± 0.28	0.61 ± 0.26*	1.03 ± 0.24***□□□	0.21 ± 0.65***□□□	0.00 ± 0.20***
PBGE-PGGE_1_CLOT_b	0.54 ± 0.13□□□	0.11 ± 0.88	0.00 ± 0.79**	26.97 ± 0.45***	0.75 ± 0.23***□□□	9.35 ± 0.34□□□
PBGE-PGGE_1_CLOT_c	6.30 ± 0.42***	0.08 ± 0.19	13.84 ± 0.14***	5.71 ± 0.07***□□□	0.11 ± 1.61***□□□	0.04 ± 0.26***
PBGE-PGGE_1_CLOT_d	23.24 ± 0.33***□□□	0.11 ± 0.77	0.11 ± 0.51***	2.63 ± 2.03***□□□	0.05 ± 0.59***□□□	5.41 ± 0.25***□□□
PBGE-PGGE_2_CLOT_a	7.07 ± 0.06***	0.08 ± 0.51	0.85 ± 0.07*	12.77 ± 0.83□□□	0.15 ± 0.77***□□□	0.03 ± 0.35***
PBGE-PGGE_2_CLOT_b	7.90 ± 0.33***	0.06 ± 0.09	0.00 ± 1.55**	6.88 ± 0.61***□□□	0.10 ± 0.66***□□□	0.13 ± 0.84***
PBGE-PGGE_2_CLOT_c	0.48 ± 0.12□□□	0.04 ± 1.24	13.10 ± 0.41***	9.07 ± 0.07***□□□	0.10 ± 0.08***□□□	0.02 ± 0.36***
PBGE-PGGE_3_CLOT_a	1.82 ± 0.30□□□	0.07 ± 2.04	0.13 ± 0.28**	12.60 ± 0.30□□□	0.15 ± 0.49***□□□	0.10 ± 0.29***
PBGE-PGGE_3_CLOT_b	4.55 ± 0.22***	0.10 ± 0.07	0.01 ± 0.50**	22.38 ± 0.09***□□□	10.04 ± 0.53***□□□	0.42 ± 0.15***
PBGE-PGGE_3_CLOT_c	4.62 ± 0.14***	0.09 ± 0.18	0.00 ± 4.10**	17.30 ± 0.12***□□□	4.07 ± 0.41***□□□	0.03 ± 0.45***
PBGE-PGGE_3_CLOT_d	10.69 ± 0.34***□□□	0.09 ± 0.89	0.15 ± 0.41**	29.05 ± 0.12***□□□	8.41 ± 0.16**□□□	0.09 ± 0.60***
**nifuratel**	**6.95 ± 0.07*****	**0.08 ± 0.13**	**0.04 ± 0.61****	**22.72 ± 0.10*****	**30.59 ± 0.14*****	**0.85 ± 0.13*****
PBGE-PGGE_1_NIF_a	5.27 ± 0.28***	0.14 ± 0.57	0.00 ± 0.93**	6.92 ± 0.64***□□□	2.48 ± 0.77***□□□	0.01 ± 0.99***
PBGE-PGGE_1_NIF_b	1.79 ± 2.28□□□	0.10 ± 0.25	0.31 ± 0.51**	4.98 ± 3.05***□□□	4.80 ± 0.24**□□□	0.03 ± 0.28***
PBGE-PGGE_1_NIF_c	5.58 ± 0.11***	0.10 ± 1.41	0.00 ± 0.46**	41.71 ± 0.21***□□□	13.91 ± 0.09***□□□	0.14 ± 0.33***
PBGE-PGGE_1_NIF_d	3.20 ± 0.97***□□□	0.05 ± 0.73	2.05 ± 0.17	1.26 ± 0.33***□□□	0.60 ± 0.46***□□□	0.66 ± 0.21***

PBGE-PGGE_1_NIF_e	5.53 ± 0.20***	0.09 ± 0.40	0.01 ± 0.15**	5.41 ± 0.64***□□□	0.60 ± 0.41***□□□	0.62 ± 0.29***
PBGE-PGGE_2_NIF_a	0.71 ± 1.77□□□	0.84 ± 0.40	0.72 ± 0.31	17.31 ± 0.32***□□□	4.56 ± 0.62**□□□	0.22 ± 0.21***
PBGE-PGGE_2_NIF_b	0.64 ± 0.36□□□	1.01 ± 0.04	0.00 ± 1.56	1.79 ± 0.01***□□□	1.67 ± 0.33***□□□	1.51 ± 0.62***
PBGE-PGGE_2_NIF_c	0.81 ± 0.48□□□	0.54 ± 1.26	3.86 ± 0.22□	10.03 ± 0.26**□□□	3.90 ± 0.27***□□□	0.21 ± 0.48***
PBGE-PGGE_2_NIF_d	1.28 ± 0.32□□□	1.18 ± 0.07	0.01 ± 0.31	6.74 ± 0.30***	3.33 ± 0.15***□□□	0.09 ± 0.27***
PBGE-PGGE_2_NIF_e	0.82 ± 0.20□□□	1.04 ± 0.27	0.14 ± 0.17	18.10 ± 0.07***□□□	4.16 ± 0.54***□□□	0.01 ± 0.07***
PBGE-PGGE_2_NIF_f	1.16 ± 0.06□□□	1.65 ± 0.01	0.00 ± 1.41	11.00 ± 0.03□□□	3.07 ± 0.84***□□□	0.02 ± 0.36***
PBGE-PGGE_3_NIF_a	0.54 ± 0.94□□□	0.90 ± 0.12	1.26 ± 0.16	14.24 ± 0.59□□□	2.72 ± 0.30***□□□	0.05 ± 0.48***
PBGE-PGGE_3_NIF_b	0.59 ± 0.71□□□	0.86 ± 0.06	0.00 ± 0.44	9.60 ± 0.45***□□□	5.59 ± 0.29□□□	0.16 ± 0.72***

After isolation of red blood cells from the blood of a healthy donor, samples with a hematocrit of 2% were incubated with the tested drugs (clotrimazole and nifuratel) and PBGE-PGGE copolyethers with/without drugs in the concentration range of 0.1 - 100 µM for 24 and 48 h, respectively. All partial results, together with the statistical analysis, are presented in two tables – hemolysis after 24 h in **Table S3** and hemolysis after 48 h in **Table S4**.

Table S3. Hemolytic activity of the tested drugs (clotrimazole and nifuratel) and PBGE-PGGE copolyethers with/without drugs in the concentration range of 0.1 - 100 µM after 24 h incubation. The results are presented as the mean ± SD, n = 4. The results were considered significant at □$p \leq 0.01$, □□$p \leq 0.001$, □□□$p \leq 0.0001$ with regard to clotrimazole and □$p \leq 0.01$, □□$p \leq 0.001$, □□□$p \leq 0.0001$ with regard to nifuratel.

	**concentration [µM]**			
	**0.1**	**1**	**10**	**100**
PBGE-PGGE_1	0.49 ± 0.33	0.89 ± 0.11	0.75 ± 0.14	1.81 ± 0.34
PBGE-PGGE_2	0.54 ± 0.15	0.54 ± 0.06	0.79 ± 0.28	0.99 ± 0.14
PBGE-PGGE_3	0.61 ± 0.15	0.50 ± 0.10	0.96 ± 0.29	1.16 ± 0.26
**clotrimazole**	**0.17 ± 0.11**	**0.07 ± 0.15**	**0.07 ± 0.07**	**0.23 ± 0.07**
PBGE-PGGE_1_CLOT_a	0.02 ± 0.09	0.31 ± 0.23	0.30 ± 0.11	0.57 ± 0.23
PBGE-PGGE_1_CLOT_b	0.18 ± 0.14	0.13 ± 0.16	0.73 ± 0.09□	3.03 ± 0.52□□□
PBGE-PGGE_1_CLOT_c	0.02 ± 0.16	0.23 ± 0.23	0.11 ± 0.13	1.30 ± 0.15□
PBGE-PGGE_1_CLOT_d	0.29 ± 0.14	0.60 ± 0.25	0.47 ± 0.19	1.93 ± 0.15□□□
PBGE-PGGE_2_CLOT_a	0.47 ± 0.23	0.42 ± 0.25	1.93 ± 0.42□□□	2.98 ± 0.65□□□
PBGE-PGGE_2_CLOT_b	0.44 ± 0.27	0.16 ± 0.12	0.19 ± 0.16	0.68 ± 0.18
PBGE-PGGE_2_CLOT_c	0.13 ± 0.11	0.34 ± 0.27	0.29 ± 0.24	1.50 ± 0.28□
PBGE-PGGE_3_CLOT_a	0.10 ± 0.07	0.12 ± 0.18	0.06 ± 0.13	1.46 ± 0.48□
PBGE-PGGE_3_CLOT_b	0.24 ± 0.08	0.10 ± 0.10	0.48 ± 0.28	1.14 ± 0.46
PBGE-PGGE_3_CLOT_c	0.08 ± 0.09	0.22 ± 0.22	0.34 ± 0.12	0.75 ± 0.21
PBGE-PGGE_3_CLOT_d	0.08 ± 0.13	0.12 ± 0.19	0.36 ± 0.44	0.43 ± 0.18
**nifuratel**	**0.10 ± 0.04**	**0.01 ± 0.11**	**0.26 ± 0.13**	**0.65 ± 0.10**
PBGE-PGGE_1_NIF_a	0.11 ± 0.31	0.13 ± 0.39	0.08 ± 0.11	0.08 ± 0.32□

PBGE-PGGE_1_NIF_b	0.05 ± 0.08	0.08 ± 0.23	0.11 ± 0.19	0.53 ± 0.16
PBGE-PGGE_1_NIF_c	0.06 ± 0.17	0.16 ± 0.16	0.13 ± 0.15	0.10 ± 0.08□
PBGE-PGGE_1_NIF_d	0.14 ± 0.10	0.08 ± 0.11	0.32 ± 0.34	0.29 ± 0.32
PBGE-PGGE_1_NIF_e	0.23 ± 0.10	0.10 ± 0.08	0.73 ± 0.11	0.90 ± 0.17
PBGE-PGGE_2_NIF_a	0.06 ± 0.09	0.24 ± 0.10	0.31 ± 0.05	0.36 ± 0.17
PBGE-PGGE_2_NIF_b	0.00 ± 0.11	0.35 ± 0.19	0.14 ± 0.14	2.26 ± 0.19□□□
PBGE-PGGE_2_NIF_c	0.11 ± 0.13	0.11 ± 0.21	0.07 ± 0.19	0.07 ± 0.07□
PBGE-PGGE_2_NIF_d	0.17 ± 0.07	0.12 ± 0.19	0.01 ± 0.12	0.55 ± 0.14
PBGE-PGGE_2_NIF_e	0.17 ± 0.11	0.14 ± 0.07	0.13 ± 0.17	0.32 ± 0.19
PBGE-PGGE_2_NIF_f	0.00 ± 0.11	0.12 ± 0.07	0.41 ± 0.20	0.42 ± 0.09
PBGE-PGGE_3_NIF_a	0.16 ± 0.12	0.24 ± 0.12	0.10 ± 0.16	0.26 ± 0.14
PBGE-PGGE_3_NIF_b	0.11 ± 0.19	0.22 ± 0.19	0.30 ± 0.13	0.20 ± 0.11

Table S4. Hemolytic activity of the tested drugs (clotrimazole and nifuratel) and PBGE-PGGE copolyethers with/without drugs in the concentration range of 0.1 - 100 μM after 48 h incubation. The results are presented as the mean ± SD, n = 4. The results were considered significant at □$p \leq 0.01$, □□$p \leq 0.001$, □□□$p \leq 0.0001$ with regard to clotrimazole and □$p \leq 0.01$, □□$p \leq 0.001$, □□□$p \leq 0.0001$ with regard to nifuratel.

	**concentration [μM]**			
	**0.1**	**1**	**10**	**100**
PBGE-PGGE_1	0.95 ± 0.09	1.34 ± 0.36	1.54 ± 0.13	1.58 ± 0.12
PBGE-PGGE_2	2.05 ± 0.25	1.20 ± 0.14	1.58 ± 0.10	1.40 ± 0.18
PBGE-PGGE_3	1.67 ± 0.45	1.48 ± 0.23	1.64 ± 0.31	1.46 ± 0.27
**clotrimazole**	**2.27 ± 0.26**	**1.66 ± 0.23**	**1.71 ± 0.09**	**2.72 ± 0.21**
PBGE-PGGE_1_CLOT_a	2.37 ± 0.15	2.28 ± 0.31	3.16 ± 0.29□□□	4.66 ± 0.22□□□
PBGE-PGGE_1_CLOT_b	1.93 ± 0.34	2.41 ± 0.32	2.47 ± 0.20□	5.38 ± 0.49□□□
PBGE-PGGE_1_CLOT_c	2.31 ± 0.64	2.10 ± 0.28	2.95 ± 0.37□□□	2.77 ± 0.28
PBGE-PGGE_1_CLOT_d	2.23 ± 0.13	2.01 ± 0.63	2.50 ± 0.21□	2.76 ± 0.24
PBGE-PGGE_2_CLOT_a	1.63 ± 0.20	1.69 ± 0.18	4.12 ± 0.36□□□	7.30 ± 0.64□□□

PBGE-PGGE_2_CLOT_b	2.37 ± 0.85	1.71 ± 0.13	1.71 ± 0.20	1.56 ± 0.07□
PBGE-PGGE_2_CLOT_c	2.29 ± 0.21	2.05 ± 0.11	2.05 ± 0.18	5.28 ± 0.31□□□
PBGE-PGGE_3_CLOT_a	2.10 ± 0.07	2.03 ± 0.19	1.89 ± 0.20	6.56 ± 0.39□□□
PBGE-PGGE_3_CLOT_b	1.82 ± 0.07	2.23 ± 0.34	2.76 ± 0.20□□	7.70 ± 0.42□□□
PBGE-PGGE_3_CLOT_c	1.30 ± 0.65	1.39 ± 0.28	1.70 ± 0.22	2.24 ± 0.40
PBGE-PGGE_3_CLOT_d	1.22 ± 0.11	1.43 ± 0.21	1.77 ± 0.21	1.56 ± 0.26□
**nifuratel**	**2.22 ± 0.30**	**1.93 ± 0.12**	**2.23 ± 0.14**	**2.58 ± 0.19**
PBGE-PGGE_1_NIF_a	1.30 ± 0.18□	1.60 ± 0.25	1.32 ± 0.25	1.64 ± 0.13□□
PBGE-PGGE_1_NIF_b	0.88 ± 0.09□□□	1.51 ± 0.07	1.50 ± 0.14	1.69 ± 0.18□□
PBGE-PGGE_1_NIF_c	0.87 ± 0.11□□□	1.16 ± 0.18	1.25 ± 0.12	1.39 ± 0.16□□□
PBGE-PGGE_1_NIF_d	0.82 ± 0.19□□□	1.15 ± 0.13	1.52 ± 0.19	2.24 ± 0.17
PBGE-PGGE_1_NIF_e	1.57 ± 0.30	1.22 ± 0.19	1.99 ± 0.32	2.68 ± 0.20
PBGE-PGGE_2_NIF_a	2.36 ± 0.45	1.48 ± 0.42	1.58 ± 0.14	1.39 ± 0.10□□□
PBGE-PGGE_2_NIF_b	1.38 ± 0.34□	1.85 ± 0.33	2.06 ± 0.29	2.84 ± 0.55
PBGE-PGGE_2_NIF_c	1.85 ± 0.17	2.02 ± 1.02	1.70 ± 0.72	1.48 ± 0.12□□□
PBGE-PGGE_2_NIF_d	1.64 ± 0.24	1.75 ± 0.35	1.76 ± 0.15	1.46 ± 0.14□□□
PBGE-PGGE_2_NIF_e	2.29 ± 0.30	1.60 ± 0.26	1.89 ± 0.36	2.40 ± 0.22
PBGE-PGGE_2_NIF_f	1.68 ± 0.12	1.77 ± 0.19	1.19 ± 0.17	1.63 ± 0.12□□
PBGE-PGGE_3_NIF_a	1.51 ± 0.24	1.82 ± 0.12	2.03 ± 0.20□	1.32 ± 0.23□□□
PBGE-PGGE_3_NIF_b	1.75 ± 0.20	1.88 ± 0.26	1.89 ± 0.31	1.16 ± 0.23□□□

To facilitate the evaluation of the microscopic images (**Figure 10** and **11**), we decided to use the ImageJ program[1] to assess the area with the cells and determine its increase or decrease caused by the action of the tested gels. Data showing the effect of the tested gels on the viability of HeLa cancer cells and non-cancerous HMEC-1 cells, immediately after gel application and after 24 hours of incubation, are presented in **Table S5**.

Table S5. Effect of pure gel (PBGE-PGGE_1), gel containing clotrimazole (PBGE-PGGE_1_CLOT_d), gel containing nifuratel (PBGE-PGGE_1_NIF_e), and gel containing equal amounts of both drugs (PBGE-PGGE_1_CLOT/NIF) on the viability of HeLa (top panel) and HMEC-1 (bottom panel) cells assayed immediately after gel administration and after 24 hours of incubation. The results were considered significant at $^{*}p \leq 0.001$ for time 24 h compared to corresponding time 0 h and □$p \leq 0.001$ with regard to control t = 24 h.

		area with cells	total area	% of area with cells relative to total area
**HeLa**	control t = 0 h	44039.00±22.16	83666.00±17.10	52.64±0.03
	control t = 24 h	57844.00±25.19	86733.00±2.17	66.69±0.03*
	pure gel PBGE-PGGE_1 t = 0 h	49752.00±25.19	86216.00±27.57	57.71±0.03
	pure gel PBGE-PGGE_1 t = 24 h	63022.00±26.75	86610.00±25.85	72.77±0.03*□
	PBGE-PGGE_1_CLOT_d t = 0 h	35453.00±23.81	86534.00±17.48	40.97±0.03
	PBGE-PGGE_1_CLOT_d t = 24 h	17544.00±24.37	86141.00±19.41	20.37±0.03*□
	PBGE-PGGE_1_NIF_e t = 0 h	45241.00±22.86	85705.00±20.52	52.79±0.03
	PBGE-PGGE_1_NIF_e t = 24 h	21748.00±31.15	86013.00±27.36	25.28±0.04*□
	PBGE-PGGE_1_CLOT/NIF t = 0 h	43878.00±23.02	87320.00±21.49	50.25±0.03
	PBGE-PGGE_1_CLOT/NIF t = 24 h	244.00±20.83	85904.00±34.96	0.28±0.02*□
**HMEC-1**	control t = 0 h	44893.00±18.78	85408.00±14.20	52.56±0.02
	control t = 24 h	73163.00±7.53	84680.00±7.26	86.40±0.01*
	pure gel PBGE-PGGE_1 t = 0 h	47970.00±19.92	86217.00±15.38	55.64±0.02

pure gel PBGE-PGGE_1 t = 24 h	69272.00±7.50	85552.00±7.37	80.97±0.01*□
PBGE-PGGE_1_CLOT_d t = 0 h	42344.00±15.36	86139.00±13.79	49.16±0.02
PBGE-PGGE_1_CLOT_d t = 24 h	40802.00±8.52	85848.00±8.45	47.53±0.01*□
PBGE-PGGE_1_NIF_e t = 0 h	42380.00±20.21	85955.00±17.52	49.30±0.02
PBGE-PGGE_1_NIF_e t = 24 h	73103.00±16.26	85556.00±18.61	85.44±0.02*□
PBGE-PGGE_1_CLOT/NIF t = 0 h	39848.00±20.48	86142.00±15.86	46.26±0.02
PBGE-PGGE_1_CLOT/NIF t = 24 h	43132.00±12.47	85264.00±12.12	50.59±0.01*□

Comparing the areas with the cells listed in **Table S5**, after 24 hours of culture, (1) the number of untreated control cells increased by 26.70% in the HeLa cancer cell line and by 64.37% in the HMEC-1 non-cancerous cell line, (2) an increase in the number of cells treated with pure PBGE-PGGE_1 gel in the HeLa line by 26.10%, in the HMEC-1 line by 45.53%, (3) a decrease in the number of cells treated with PBGE-PGGE_1 hydrogel enriched with clotrimazole in the HeLa line by 50.29%, in the HMEC-1 line by 3.31%, (4) decrease in the number of cells treated with hydrogel based on PBGE-PGGE_1 enriched with nifuratel in the HeLa line by 52.10%, increase in the HMEC1 line by 73.30%, and (5) decrease in the number of cells treated with hydrogel based on PBGE-PGGE_1 enriched with both drugs in the HeLa line by 99.43%, increase in the HMEC-1 line by 9.36%, demonstrating (i) no inhibitory effect of the pure gel on cell growth, (ii) the selective effect of the gel formulation enriched with clotrimazole, nifuratel and both drugs, and (iii) the increased efficacy of the gel enriched with both drugs compared to gels containing single drugs.

Table S6. Effect of pure gel (PBGE-PGGE_2), gel containing clotrimazole (PBGE-PGGE_2_CLOT_c), gel containing nifuratel (PBGE-PGGE_2_NIF_d), and gel containing equal amounts of both drugs (PBGE-PGGE_2_CLOT/NIF) on the viability of HeLa (top panel) and HMEC-1 (bottom panel) cells assayed immediately after gel administration and after 24 hours of incubation. The results were considered significant at *p ≤ 0.001 for time 24 h compared to corresponding time 0 h and □p ≤ 0.001 with regard to control t = 24 h.

		area with cells	total area	% of area with cells relative to total area
**HeLa**	control t = 0 h	47656.00±12.43	87616.00±16.87	54.39±0.02
	control t = 24 h	81144.00±12.47	87024.00±18.62	93.24±0.02*
	pure gel PBGE-PGGE_2 t = 0 h	48334.00±29.15	87616.00±25.94	55.17±0.03
	pure gel PBGE-PGGE_2 t = 24 h	74961.00±20.61	87024.00±20.48	86.14±0.02*□
	PBGE-PGGE_2_CLOT_c t = 0 h	40415.00±18.63	87025.00±17.49	46.44±0.02
	PBGE-PGGE_2_CLOT_c t = 24 h	31464.00±16.47	86730.00±14.80	36.28±0.02*□
	PBGE-PGGE_2_NIF_d t = 0 h	10585.00±12.57	86435.00±8.72	12.25±0.01
	PBGE-PGGE_2_NIF_d t = 24 h	436.00±5.39	85992.00±15.13	0.51±0.01*□
	PBGE-PGGE_2_CLOT/NIF t = 0 h	39690.00±13.46	86436.00±10.05	45.92±0.02
	PBGE-PGGE_2_CLOT/NIF t = 24 h	79.00±38.27	86730.00±14.20	0.09±0.04*□
**HMEC-1**	control t = 0 h	45135±14.82	87320±12.30	51.69±0.02
	control t = 24 h	85756±11.47	87024±11.51	98.54±0.01*
	pure gel PBGE-PGGE_2 t = 0 h	42924±13.79	86436±13.38	49.66±0.02
	pure gel PBGE-PGGE_2 t = 24 h	86140±10.66	86435±10.66	99.66±0.01*□
	PBGE-PGGE_2_CLOT_c t = 0 h	38480±17.64	87320±14.42	44.07±0.02
	PBGE-PGGE_2_CLOT_c t = 24 h	34747±11.60	86140±10.90	40.34±0.01*□
	PBGE-PGGE_2_NIF_d t = 0 h	29304±17.33	87024±12.90	33.67±0.02
	PBGE-PGGE_2_NIF_d t = 24 h	28036±7.89	86730±7.72	32.33±0.01*□
	PBGE-PGGE_2_CLOT/NIF t = 0 h	36162±11.62	86436±10.28	41.84±0.01
	PBGE-PGGE_2_CLOT/NIF t = 24 h	35269±9.72	86436±9.41	40.80±0.01*□

Comparing the areas with the cells listed in **Table S6**, after 24 hours of culture, (1) the number of untreated control cells increased by 71.43% in the HeLa cancer cell line and by 90.65% in the HMEC-1 non-cancerous cell line, (2) an increase in the number of cells treated with pure

PBGE-PGGE_1 gel in the HeLa line by 56.14%, in the HMEC-1 line by 100%, (3) a decrease in the number of cells treated with PBGE-PGGE_1 hydrogel enriched with clotrimazole in the HeLa line by 21.88%, in the HMEC-1 line by 8.46%, (4) decrease in the number of cells treated with hydrogel based on PBGE-PGGE_1 enriched with nifuratel in the HeLa line by 95.86%, in the HMEC1 line by 8.46%, and (5) decrease in the number of cells treated with hydrogel based on PBGE-PGGE_1 enriched with both drugs in the HeLa line by 99.80%, in the HMEC-1 line by 2.47%, demonstrating (i) no inhibitory effect of the pure gel on cell growth, and (ii) the selective effect of the gel formulation enriched with clotrimazole, nifuratel and both drugs.

[S1] Schneider CA. Rasband WS. Eliceiri KW. NIH Image to ImageJ: 25 years of image analysis. Nature Methods. 2012 Jul;9(7):671-5. doi: 10.1038/nmeth.2089
